# Revisiting Hansen Solubility Parameters by Including Thermodynamics

**DOI:** 10.1002/cphc.201700408

**Published:** 2017-10-06

**Authors:** Manuel J. Louwerse, Ana Maldonado, Simon Rousseau, Chloe Moreau‐Masselon, Bernard Roux, Gadi Rothenberg

**Affiliations:** ^1^ Van‘t Hoff Institute for Molecular Sciences University of Amsterdam, Postbus 94720 1090 GS Amsterdam The Netherlands; ^2^ Inorganic Chemistry and Catalysis Debye Institute for Nanomaterials Science Utrecht University Universteitsweg 99 3584 CG Utrecht The Netherlands; ^3^ Solvay, Research & Innovation Center 178 Av. Dr Albert Schweitzer F-33608 Pessac France; ^4^ Solvay, Research & Innovation Center Paris/Novecare 52 rue de la haie Coq F-93308 Aubervilliers France

**Keywords:** donor–acceptor interactions, entropy, solubility parameters, temperature, thermodynamics

## Abstract

The Hansen solubility parameter approach is revisited by implementing the thermodynamics of dissolution and mixing. Hansen's pragmatic approach has earned its spurs in predicting solvents for polymer solutions, but for molecular solutes improvements are needed. By going into the details of entropy and enthalpy, several corrections are suggested that make the methodology thermodynamically sound without losing its ease of use. The most important corrections include accounting for the solvent molecules’ size, the destruction of the solid's crystal structure, and the specificity of hydrogen‐bonding interactions, as well as opportunities to predict the solubility at extrapolated temperatures. Testing the original and the improved methods on a large industrial dataset including solvent blends, fit qualities improved from 0.89 to 0.97 and the percentage of correct predictions rose from 54 % to 78 %. Full Matlab scripts are included in the Supporting Information, allowing readers to implement these improvements on their own datasets.

## 1 Introduction

Solvents are extremely important in many industrial sectors. As chemists, we tend to think mainly about chemical reactions and active ingredients. But in real‐world terms, the solvent and formulation of a chemical product, be it an ink, a paint, an oil derivative, or a herbicide, makes the bulk of the product. Often, it is also crucial for the function of the product: the formulation is what makes a pesticide stay longer on the leaves after rain, determines the distribution and drying of paints and inks, or controls the applicability of health and cosmetics products. Hence, knowing the solubility (or lack thereof) of chemicals is of utmost importance.

Moreover, when selecting a solvent, usually not only its ability to dissolve the active ingredient matters, but many other constraints apply as well (e.g. viscosity, volatility, sustainability, and safety of the formulation). Therefore, to efficiently search for satisfying formulations, a predictive tool for solubility is indispensable.^1^, ^2^

The paint and coatings industry, for example, has been using solubility parameters for many decades.^3^ These are simple parameters for solvents and solutes based on the “like dissolves like” concept: when a solute and a solvent make similar interactions with each other as with their own kinds, there is no or little enthalpy loss upon mixing. The first solubility parameters were suggested by Hildebrand, who used a single parameter, *δ*, based on the cohesive energy per volume [Eq. (1)]:^4^

(1) $$\delta =\sqrt{\frac{E_{\mathrm{cohesion}}}{V_{\mathrm{mol}}}}$$

where *E*_cohesion_=Δ*H*_vap_−*RT*.

As long as the interactions are purely dispersive, for example, hydrocarbons versus fluorinated compounds, Hildebrand's method is reasonably accurate. However, for more general cases, it was Hansen who realized in the 1960s that the cohesive energy and the corresponding solubility parameters should be split into the three fundamental chemical interactions: dispersion (D), polar interactions (P), and hydrogen bonding (H) [Eqs. (2), (3)]:^5^, ^6^, ^7^

(2) $$E_{\mathrm{cohesion}}=E_{D}+E_{P}+E_{H}$$

(3) $$\delta^{2}={\delta_{D}}^{2}+{\delta_{P}}^{2}+{\delta_{H}}^{2}$$

**Figure 1:**
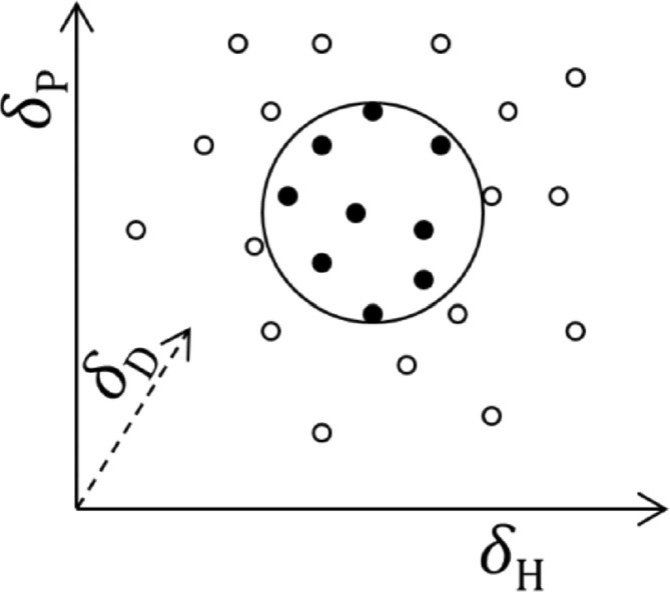
Three‐parameter plot showing the solubility sphere in the original Hansen method. Solvents are plotted according to their solubility parameters, *δ*_D_, *δ*_P_, and *δ*_H_. For each solute, a sphere is fitted according to its solubility in these solvents. The black dots denote the solvents that dissolve the solute.

Albeit indirectly, Hansen measured the contribution of these three interaction types to the cohesive energy for many solvents. For solutes, the solubility parameters are determined by measuring the solubility at a given concentration in a set of solvents. To do this, one first plots the solvent parameter values in a three‐dimensional space. Then, a sphere is fitted for the solute corresponding to the measured data (see Figure 1). The radius of this sphere gives a fourth parameter that is specific for this solute and depends on the temperature and the concentration. Now, the solubility in other solvents at this temperature and concentration can be predicted by checking whether the parameters of the new solvent fall inside or outside the sphere. Conveniently, for solvent blends the parameters can be calculated by a simple linear interpolation based on the volume/volume ratio of the blend. When materials do not actually dissolve, swelling experiments^6^, ^8^ or inverse gas chromatography^9^, ^10^ may also be performed to find the Hansen parameters.

Hansen's methodology was first applied in the paint and polymer industries and was found to be satisfactory. More recently, Hansen parameters have also been used in other sectors, to find solvents for all types of (small) molecules, including drugs,^11^ cosmetics,^12^ and oligomers,^13^ as well as for predicting gel formation.^14^, ^15^, ^16^ However, the results are not as good when compared with those for polymers. There are two reasons for this: the first is that drugs, cosmetics, and the like typically have more varied functional groups. The second reason is that the original Hansen parameters do not include thermodynamic considerations. This is acceptable for polymers (where the thermodynamics cancel out) but not for small molecule solutes.

Some rigorous thermodynamic derivations were published by Coleman et al. proving the correctness of Hildebrand's method for nonpolar polymers and extending a Hildebrand/Hansen intermediate (including polar interactions, but excluding hydrogen‐bonding interactions) to nanotubes and nanosheets.^17^ However, thermodynamic corrections for small molecule effects and a correct handling of hydrogen‐bonding interactions are still missing.

Here, we revisit the Hansen solubility parameter method for small molecules by adding thermodynamics to it. We introduce several fundamental corrections that improve the quality of the predictions. Our goal is to keep the conceptual simplicity of Hansen's method. Therefore, we refrain from including quantities that are difficult to measure, such as heats of melting or heat capacities. The new model is tested on a large industrial dataset, and shows a significant improvement over the original Hansen method.

## 2 Theory

We present five improvements to Hansen's method, following from the actual thermodynamics of dissolution and mixing. When a compound dissolves, molecules leave the crystal and mix into the solvent. This gives a positive entropy effect, but usually costs some enthalpy. This enthalpy loss is related to the distances in the Hansen plots. The key issue here is that the amount of entropy gained by mixing determines how much enthalpy can be lost while maintaining a negative Δ*G*. The distance that gives Δ*G*=0 defines the radius of the Hansen sphere. As the entropy effect depends on the concentration, the temperature, and the size of the molecules, this should all be included in the methodology. Our improvements are based on a better description of both the entropy and the enthalpy terms.

Note that the term “solubility spheres” is used; yet these spheres refer only to the parameter space (see Figure 1) and not to physical radii of molecules or other species.

### 2.1 Improvements Related to Entropy

#### 2.1.1 #1 Introducing Solvent Radii

Assuming regular mixing, the entropy of mixing per mole of total material (solute plus solvent) is [Eq. (4)]:^18^

(4) $$\Delta S_{\mathrm{mix}}=-R\left[x\ln x+\left(1-x\right)\ln\left(1-x\right)\right]$$

Where *x* is the mole fraction of the solute and *R* is the ideal gas constant. As this is per mole, solvents with small molecules give a higher entropy of mixing per liter of solvent (and are indeed known as better solvents). In the original Hansen method, however, all solvents are considered equally good, and solubility only depends on the parameter distance from the solute.

**Figure 2:**
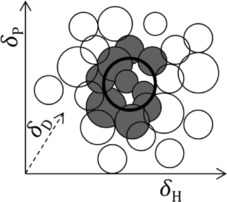
Schematic showing the philosophy behind including solvent radii: the allowed distance now also depends on the solvent radius. Note that using the inverse summation (and other non‐linear corrections discussed below) precludes any further graphical data analysis.

Here, we introduce radii (in the parameter space) for the solvents as well. These would be inversely correlated to the solvents’ molar volume. Figure 2 shows the concept of this improvement using the sum of the radii of the solute and the solvent as cutoff. In practice, we propose using the *inverse* sum of these radii [Eq. (5)]:

(5) $$r_{\mathrm{eff}}=\frac{1}{\frac{1}{r_{\mathrm{solute}}}+\frac{1}{r_{\mathrm{solvent}}}}$$

The rationale behind choosing the inverse sum is that it makes the smaller value more important: if the solute is notoriously difficult to dissolve, it has a small parameter radius and the effective radius should be small as well; if the solute is for instance a solvent itself (with a large parameter radius), the effective radius should be more equally weighted between the solute's and the solvent's radii.

#### 2.1.2 #2 Correcting for Concentration

Usually, Hansen spheres are fitted to data for one concentration. However, as established above, the entropy depends on the mole fraction, and this may differ nonetheless as a result of the different molar volumes of each solvent. Hence, we must correct for combining data at different mole fractions into one fit.

The radius of the Hansen sphere is defined by the ratio between entropy and enthalpy of mixing [Eq. (6)]:

(6) $$\Delta H_{\mathrm{mix}}=-\alpha \beta x\left(1-x\right)$$

where *β* is the so‐called interaction parameter, and *α* is the activity coefficient caused by non‐ideal mixing (*α*=1 for regular mixtures). If we now define the Hansen radius for a certain benchmark concentration, for example, *x=*0.5 [Eq. (7)], we can calculate the radius at any other concentration by correcting for the change in the ratio between Δ*S* and Δ*H* [Eqs. (4) and (6)]:(7)
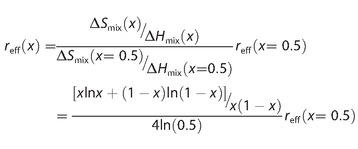



An additional advantage of this correction is that we may now combine data for different concentrations.

### 2.2 Improvements Related to Enthalpy

#### 2.2.1 #3 Using the Squared Distance

As derived by Coleman et al.^17^ and already mentioned by Hansen,^19^ the enthalpy of mixing relates to the *square* of the parameter distance: the interaction parameter, *β*, is [Eq. (8)]:

(8) $$\beta =-\frac{1}{2}\left[{\left(\delta_{D,A}-\delta_{D,B}\right)}^{2}+{\left(\delta_{P,A}-\delta_{P,B}\right)}^{2}+{\left(\delta_{H,A}-\delta_{H,B}\right)}^{2}\right]$$

where the subscripts A and B represent solute and solvent parameters, respectively. Nevertheless, Hansen uses the unsquared distance to optimize the fit. In principle, this does not matter as squaring the radius does not affect a sphere's shape. It only influences the weight of points that are missed by the fit. However, now that we have introduced solvent spheres, a different *r*_eff_ applies for every solvent. Thus, now it does matter whether *r*_eff_ or *r*_eff_^2^ is used, so we stress that Equation (8) should be used and not its square root.

#### 2.2.2 #4 Splitting Donor and Acceptor Parameters

For hydrogen bonding, the reasoning “like dissolves like” is imprecise: hydrogen bonds form between donors and acceptors, so to dissolve donors one needs acceptors, and vice versa. Therefore, the *δ*_H_ parameter needs to be split into a *δ*_HD_ (donor) and a *δ*_HA_ (acceptor) parameter. Now, the cohesive energy contribution of hydrogen bonds (or any other Lewis acid–base interactions) is defined as [Eq. (9)]:

(9) $$E_{\mathrm{cohesion},H}=\delta_{\mathrm{HD}}\delta_{\mathrm{HA}}$$

A recent version of the HSP software (v4.0, 2013) already attempts to include this effect, giving moderate improvements. There, the definition of *δ*_HD_ and *δ*_HA_ was inspired by the work of Abraham, including the reasoning that Equation (9) does not allow for a non‐zero *δ*_H_ combined with a zero *δ*_HD_.^6^, ^20^ Hence, they defined the relation differently: *δ*_H_^2^=*δ*_HD_^2^+*δ*_HA_^2^. But if *δ*_H_ is not equal to zero, then *δ*_HD_ (or *δ*_HA_) cannot be zero either: if the experimental cohesive energy shows that there are non‐zero Lewis acid–base interactions in the pure substance, then the molecules must have both Lewis acid *and* base character (even if chemical intuition suggests they do not).

Following from Equation (9), the hydrogen‐bonding contribution to the interaction parameter, *β*, becomes [Eq. (10)]:

(10) $$\beta_{H}=\frac{1}{2}\left(\delta_{\mathrm{HD},A}\delta_{\mathrm{HA},B}+\delta_{\mathrm{HD},B}\delta_{\mathrm{HA},A}-\delta_{\mathrm{HD},A}\delta_{\mathrm{HA},A}-\delta_{\mathrm{HD},B}\delta_{\mathrm{HA},B}\right)$$

In this way, we recognize the advantageous mixing of donor species with acceptor species, in which case *β* can even become positive. However, split donor and acceptor parameters for the solvents are very hard to obtain experimentally, as they are coupled. Following Hunter, though, they can be calculated from the electrostatic potential in electronic structure calculations.^21^ Hunter compiled a table with donor and acceptor strengths of many functional groups. But as we do not want to include specific knowledge of the number of functional groups in each solvent or solute molecule, we need volume‐based parameters. Therefore, for each solvent, we took the average of the values of all the functional groups in the molecule and scaled this to fit with the known value for *δ*_H_ (see the Supporting Information for details). This averaged approach is approximate, yet it works reasonably well (cf. the work of Beerbower et al., who estimated Lewis acid and base components based on spectroscopic measurements^22^).

### 2.3 Other Improvements

#### 2.3.1 #5 Including Enthalpy and Entropy of Melting

Many solutes are solid at room temperature. This means that upon dissolution, there are not only enthalpy and entropy effects of the mixing itself, but also of the destruction of the crystal structure. Thus, the enthalpy loss of melting and mixing needs to be overcome by the entropy gain of melting and mixing. Note that these are the virtual enthalpy and entropy of melting at the mixing temperature, which may differ from those at the melting point owing to differences in heat capacity.^23^ Putting this into equations, we should realize that the enthalpy and entropy of melting are defined per mole of solute, whereas the mixing quantities are defined per total number of molecules [Eq. (11)]:

(11) $$T\left(\Delta S_{\mathrm{mix}}+x\Delta S_{\mathrm{melt},T}\right)\geq \Delta H_{\mathrm{mix}}+x\Delta H_{\mathrm{melt},T}$$

On the limit of solubility both sides are equal, and Equation (12) applies:

(12) $$\Delta H_{\mathrm{mix}}=T\Delta S_{\mathrm{mix}}-x\left(\Delta H_{\mathrm{melt},T}-T\Delta S_{\mathrm{melt},T}\right)$$

We now replace $\left(\Delta H_{\mathrm{melt},T}-T\Delta S_{\mathrm{melt},T}\right)$ with a constant, *c*_melt_, and, because the Hansen radius is related to *β* instead of $\Delta H_{\mathrm{mix}}$ , we divide by *x*(1−*x*) [see Eq. (13)]:

(13) $$r_{\mathrm{eff}}=\frac{T\Delta S_{\mathrm{mix}}}{x(1-x)}-\frac{c_{\mathrm{melt}}}{1-x}$$

The first part of this equation is not influenced by the solute melting, and can be replaced by Equation (5) [or Eq. (7)] to give Equation (14):

(14) $$r_{\mathrm{eff}}=\frac{{}^{'}\mathrm{concentration}\_\mathrm{correction}{}^{'}}{\frac{1}{r_{\mathrm{solute}}}+\frac{1}{r_{\mathrm{solvent}}}}-\frac{c_{\mathrm{melt}}}{1-x}$$

We can thus correct for the melting of the solute by subtracting a simple (temperature‐dependent) constant, *c*_melt_ divided by (1−*x*). The constant *c*_melt_ is the free energy lost by breaking down the crystal structure of the solid solute. However, its value is unknown (because of the aforementioned heat capacity effects) and needs to be optimized while fitting the data. Moreover, owing to the same effects, *c*_melt_ depends unpredictably on *T*. In principle, though, *c*_melt_ should always be positive, because $\Delta H_{\mathrm{melt}}$ is larger than $T\Delta S_{\mathrm{melt}}$ at any *T* below the melting point.

#### 2.3.2 The Effect of Temperature

As we now have better descriptions of entropy and enthalpy, we can also predict their dependence on temperature. For moderate temperature differences, we can assume the solubility parameters, *δ*_D_, *δ*_P_, and *δ*_H_, to be constant. This assumption is not valid for large temperature differences, though:^7^ in the liquid state, at higher temperatures the molecules will rotate more, giving less optimal polar interactions, and hydrogen bonds will break more often. As a result, the cohesive energy resulting from polar (*δ*_P_) and hydrogen‐bonding (*δ*_H_) interactions will decrease. This effect can be estimated by calculating the Boltzmann weighted averages over the molecular orientations of polar compounds and over the number of broken hydrogen bonds.^24^

Note, however, that the same effect occurs in all the liquid states: pure solvent, molten solute, and solution. The major relative effect is to be expected for the solid solute, as in the solid phase rotational freedom and broken hydrogen bonds are less frequent. However, this difference goes into the *c*_melt_ parameter and not into the solubility parameters, *δ*. Hence, we believe that the relative effect (besides a formal shift for all solvents and solutes) of temperature on the solubility parameters, should be small, and will depend on the actual combination of strongly and weakly binding functional groups in a specific solute–solvent pair.

Within the approximation of regular mixing, neither the entropy nor the enthalpy of mixing depend on the temperature. Therefore, the only effect is the dependence of the Gibbs free energy itself: Δ*G*=Δ*H*−*TΔS*. As the solubility radius is essentially the (corrected) distance in the parameter space for which Δ*H*=*T*Δ*S* (i.e. Δ*G*=0), all solubility radii (*r*_solute_ and *r*_solvent_) depend linearly on *T* [Eq. (15)]:

(15) $$r\left(T\right)=\frac{T}{T^{0}}r^{0}$$

That said, there is one complication: the *c*_melt_ constant introduced in Equation (13) also depends on the temperature. If the heat capacity of the solid is constant with varying *T*, *c*_melt_ would depend linearly on *T*, but relative to the melting point, *T*_M_ [Eq. (16)]:

(16) $$c_{\mathrm{melt}}\left(T\right)=\frac{T_{M}-T}{{T_{M}-T}^{0}}c_{\mathrm{melt}}^{0}$$

In practice, the heat capacity varies and tends to increase strongly close to the melting point. As a result, for some solutes, when far from the melting point, it may be better to consider *c*_melt_ to be constant with *T*, which follows from substituting *T*_M_=∞ into Equation (16) to give Equation (17):

(17) $$c_{\mathrm{melt}}\left(T\right)=c_{\mathrm{melt}}^{0}$$

As the behavior of the heat capacity is very compound‐specific, choosing between Equations (16) and (17) is difficult. Therefore, we pragmatically suggest using the average of these two equations [Eq. (18)]:

(18) $$c_{\mathrm{melt}}\left(T\right)=\frac{1}{2}\left(\frac{T_{M}-T}{{T_{M}-T}^{0}}+1\right)c_{\mathrm{melt}}^{0}$$

For Equations (16) and (18), the melting point of the solute is needed. However, as it is unclear which equation is the best to use in the first place, an estimate of the melting point suffices.

#### 2.3.3 Interpolation of Parameters for Blends

An important advantage of the Hansen methodology is that it can easily predict solubilities in solvent blends as well. It is common practice to estimate the parameters for such blends by linear interpolation of the parameters of the constituting pure solvents. Now that we have improved the method from pragmatically effective to thermodynamically sound, we can check the correctness of such interpolations. Also, we need mixing rules for the new parameters that we introduced: *r*_solvent_, *δ*_HD_, and *δ*_HA_. Writing down the full derivations (see the Supporting Information) shows that the correct mixing rule is indeed approximately a linear interpolation based on the volume/volume ratio of the solvents in the blend. In particular, we find that the same mixing rule applies for all parameters.

#### 2.3.4 Concerning the Correction Factor for δ_*D*_

In the original Hansen solubility methodology, all *δ*_D_ values are multiplied by an additional empirical factor of 2, simply because this improves the agreement with experimental data.^5^, ^6^, ^7^ However, until today no physical explanation was found for this correction factor.^17^ Incidentally, above we explained that temperature‐dependent Boltzmann weighing of molecular orientations will lower the effective *δ*_P_ and *δ*_H_ values. This might be the physical explanation of Hansen's correction factor. However, one should realize that this effect depends highly on the strength of specific interactions and on the actual temperature. A constant correction factor of 2 is surely unphysical.

## Methods

We tested our improved methodology on an industrial dataset of 15 solutes of very different types, including herbicides, organometallic complexes, electrolytes, polymers, and other compounds. The solutes’ solubility parameters were determined by measuring their solubility at fixed concentrations in a standard set of 48 solvents (with parameters ranging from 12.9–21.0 for *δ*_D_, 0–26.2 for *δ*_P_, and 0–42.3 for *δ*_H_). Some of the solutes were tested at multiple concentrations, resulting in a total of 21 fit sets. Experiments in other randomly chosen solvents (172 data points) and solvent blends (284 data points) were used as a prediction set. All experiments were performed at room temperature by a liquid handling robot using 1.00 mL solvent per vial, and the degree of dissolution (complete or incomplete) was determined visually after mixing for 24 h.

For four out of the 15 solutes, we also ran experiments at lower temperatures, ranging from 4 °C down to −10 °C. These samples were first mixed at room temperature. After mixing for 24 h, they were cooled for 24 h; then a crystalline seed of the solute was added followed by another 24 h of cooling, before the degree of dissolution was determined. In total, four fit sets and a prediction set of 125 data points were collected at lowered temperatures.

Before being able to fit the solute parameters, a priori values for the solvents are needed for the new parameters, *r*_solvent_, *δ*_HD_, and *δ*_HA_. The radius *r*_solvent_ was assumed to be inversely proportional to the molar volume, *V*_M_, and was calibrated on solvent–solvent mixing data, resulting in Equation (19):

(19) $$r_{\mathrm{solvent}}\left(x=0.5\right)\approx \frac{1600\sqrt{J\;\mathrm{mL}}/\mathrm{mol}}{V_{M}}$$

When comparing partly improved methodologies, *r*_solvent_ was calibrated for each choice of settings. The parameters *δ*_HD_ and *δ*_HA_ were estimated as described in the Supporting Information and were scaled to fit with the known values for *δ*_H_. Furthermore, for calculating the mole fraction, *x*, of a solute its molar mass needs to be known. For polymers, any large number can be used, as for large masses the actual value makes little difference; we used 10^10^ g mol^−1^ (we checked that using 10^4^ g mol^−1^ gives practically the same results, but if the molar mass is known it is clearly best to use the true value). Note that *c*_melt_ only applies to solutes, so no a priori values are needed.

The fitting was performed by steepest‐descent optimization of *δ*_D_, *δ*_P_, and *δ*_H_, combined with stepwise adjustment of *r*, *c*_melt_, and the *δ*_HD_/*δ*_HA_ ratio. However, owing to the Boolean nature of the data (complete vs. incomplete dissolution) the optimized function has discontinuous derivatives, making robust optimization difficult. To overcome this, we performed optimizations from a concentric grid of 129 starting points around the average of all good solvents and selected the solution with the best fit, usually leading to better fits than with the HSP software. In the case of perfect fits, the average was taken of all perfect solutions found. For fair comparison, the same algorithm was used both for testing the original Hansen method and our improved methodology.

Note that the fitting procedures for Hansen spheres are themselves a subject of discussion.^25^, ^26^, ^27^ Moreover, the usual definition of the optimum is problematic: when two data points are misfitted at opposite sides of the Hansen sphere, any solution between these two points gets the same score. Improvements of the fitting procedure itself should also focus on this linear definition of the optimum, but this is outside the scope of this work. More details of our fitting procedure as well as the script for implementing it are included in the Supporting Information.

The quality of the fit, *q*_F_, was calculated as proposed by Hansen:^19^ for data points at the wrong side of the sphere, the error is calculated by calculating the distance of this point to the edge of the sphere (or actually by calculating the difference between the interaction parameter *β* and the value for *β* that would place the data point exactly on the edge of the sphere). Then, *q*_F_ is calculated as follows [Eq. (20)]:(20)
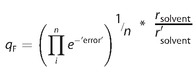



Here, *n* is the total number of data points in the fit (not only the ill‐fitted points). A perfect fit gets a value of 1; non‐perfect fits get lower values. Note that the values are corrected for the absolute scale of the radii, where *r*_solvent_ is any solvent's radius calibrated for the original method and *r′*_solvent_ is the equivalent radius calibrated for a given set of improvements. In fact, for the fully improved method the calibrated scale is very similar to that of the original method.

## 3 Results

**Figure 3:**
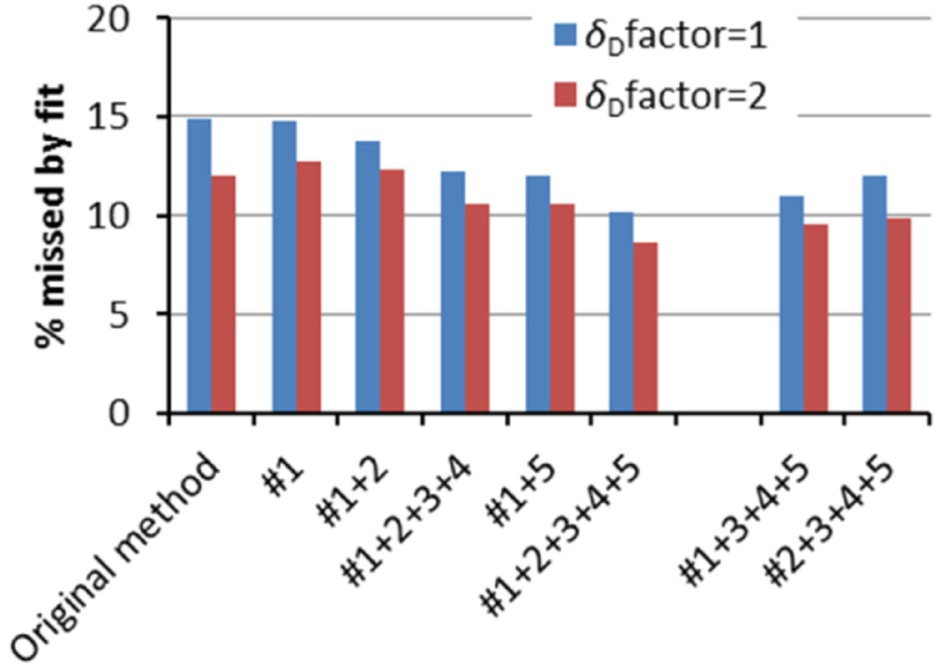
Numbers of missed data points during fitting of 21 training sets with our improved method versus Hansen's original method, comparing the effect of separate improvements. #1+2+3+4+5 denotes the fully improved method. *δ*_D_factor=2 refers to the correction factor Hansen uses for the *δ*_D_ values; our tests were performed with and without this factor.

**Figure 4:**
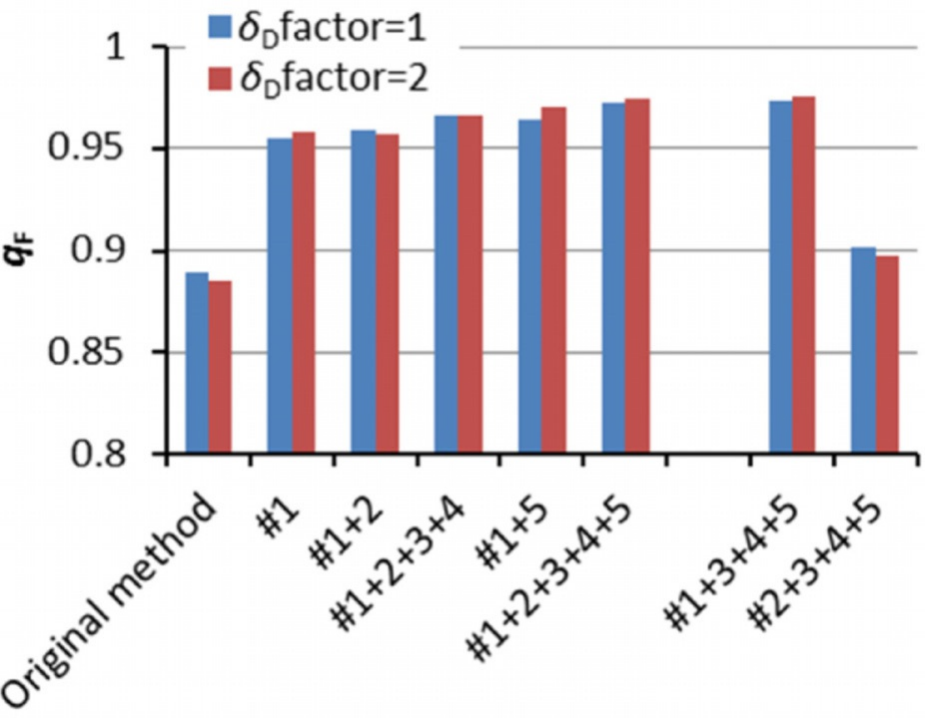
Average *q*_F_ (fit quality) for 21 training sets with our improved method versus Hansen's original method. Perfect fits would give *q*_F_=1.

To establish the power of Hansen's original method and our improved method, we assessed both the ability to fit known data as well as the quality of predictions for unseen solvents based on these fits. Also, the relative importance of the various theoretical improvements was assessed by testing subsets, where the numbers refer to the numbering in the theory section. Note that improvements #3 and #5 are effective only when improvements #1 or #2 are used. Similarly, improvement #4 only makes sense in combination with #3. Figures 3 and 4 show the ability to fit known data, expressed by the percentage of data points that could not be fitted at the correct side of the sphere, and by the fit quality, *q*_F_. Although the improvement in the number of missed points is modest, *q*_F_ improves impressively, which means that points that could not be fitted, still are approached much closer.

**Figure 5:**
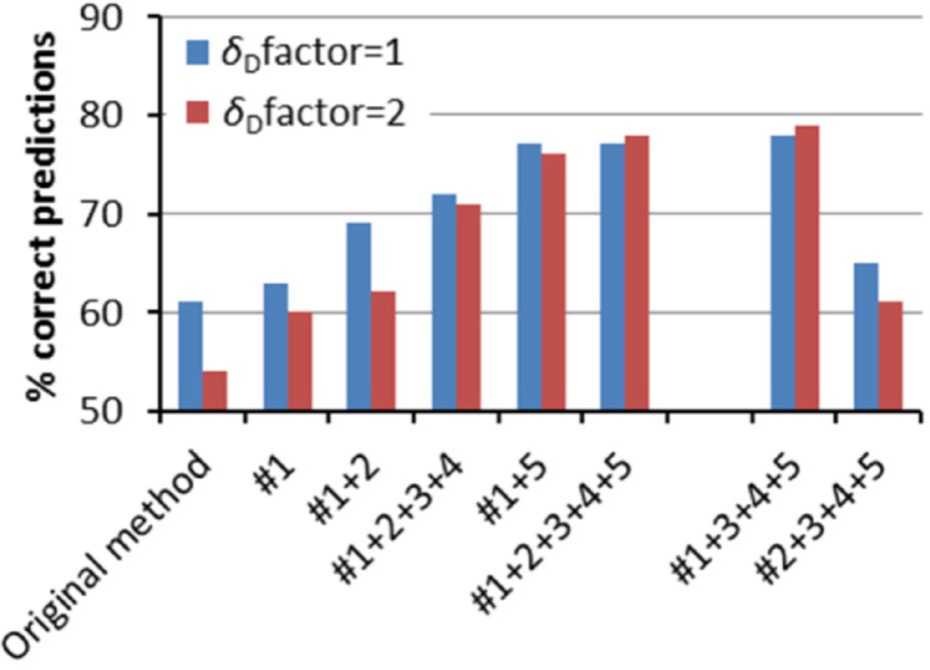
Results for the prediction of solubility at room temperature in unseen solvents and solvent blends (see Methods section) with our improved methods versus Hansen's original method. Note that, owing to the Boolean nature of the data, 50 % correct predictions means zero predictive value.

As high fit scores may always be caused by overfitting,^28^ the most important results are the predictions of the solubility in unseen solvents and solvent blends (Figure 5). These are strongly improved as well. Interestingly, improvements #1 and #5 apparently are the most important. The combination of only these two improvements already leads to a *q*_F_ of 0.96 and 77 % correct predictions. With respect to the *δ*_D_ correction factor, we note that, although the percentage of missed data points during fitting becomes better when the factor is included, the quality of the predictions actually gets worse. Interestingly, we found that the predictions are even more improved for solvent blends than for pure solvents. However, as this refers to different data points, such numbers cannot be compared. Therefore, we did not analyze the difference between pure solvents and blends.

Overall, our complete improved method (improvements #1+2+3+4+5) leads to an improvement of the *q*_F_ going from 0.89 for Hansen's original method to 0.97, which is almost four times closer to perfect fitting (*q*_F_=1). Moreover, the percentage of correct predictions improved from 54 % to 78 %. Realizing that random predictions will also be correct for 50 % of the points, the improvement is large.

### 3.1 Predicting the Solubility at Extrapolated Temperatures

**Figure 6:**
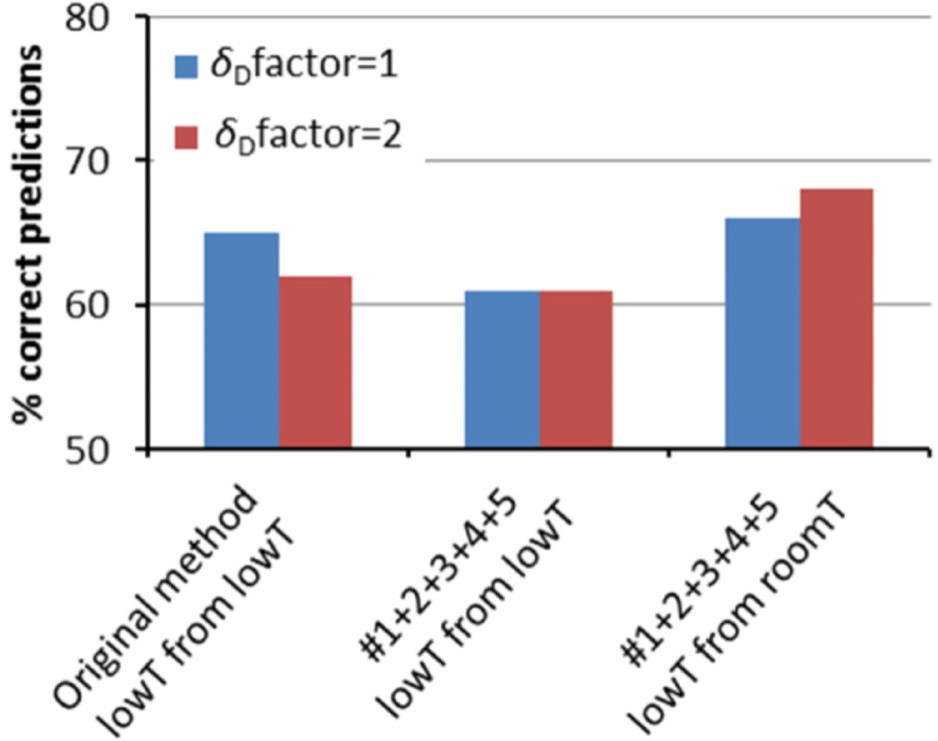
Prediction of solubility at low temperatures (4 °C to −10 °C). “low *T* from low *T*” means that training sets and prediction sets are obtained at the same temperature. “Low *T* from room *T*” means that the same data set is predicted from training sets obtained at room temperature, using the opportunity to extrapolate the temperature in the improved method.

The dependences of the solubility parameters, including *r* and *c*_melt_, on the temperature, allow predictions to be made at extrapolated temperatures. This was tested by predicting solubilities at lowered temperatures based on experimental tests at room temperature. For comparison, the solubility in the standard test set of 48 solvents was also measured at the lowered temperature for predictions without extrapolation. Note that the percentages of correct predictions are lower than in Figure 5. This is because only a few of the standard solvents dissolve the solutes at lowered temperature, resulting in inaccurate fitted spheres. Although the statistics for these tests are rather limited, Figure 6 shows that the extrapolated predictions are at least as good as the predictions at constant temperature. In fact, the extrapolated predictions are better. The latter reflects the fact that at higher temperatures more solvents can dissolve a given solute, which gives more information for fitting and thus a more precise fit. Note that this improvement for the extrapolated predictions is statistically relevant, whereas the difference between the original method and the improved method is not statistical relevant for this (small) low *T* dataset.

## 4 Discussion

Methods for predicting solubility are typically very generalized to make predictions for new solutes and unseen solvents. On the other hand, enough accuracy is needed to be of practical value. We show that by considering the thermodynamics of solubility, simple generalized models can be improved significantly without sacrificing the general applicability. That said, generalized methods are never exact. For instance, most solubility models assume regular mixing, but reality is full of effects that break ideality that cannot be accounted for in a theoretical framework. For this reason, we stuck to the Hansen concept of fitting, as this should capture at least part of these non‐idealities without incorporating them explicitly.

In recent years, several other models have been used for predicting solubility fully theoretically. Examples are: UniQuac,^29^ COSMO‐RS,^30^ and even a combination of COSMO and Hansen.^31^ However, COSMO models only aim at improving the description of the enthalpy terms. Therefore, these methods could also be improved further by including entropy and melting corrections, similarly to what we have shown here. We do note that fully theoretical methods will be haunted by non‐idealities to a larger extent than a procedure of fitting to real experimental data such as the Hansen methodology. Hansen parameters can also be estimated theoretically, as studied extensively by Panayiotou et al.^32^, ^33^ They even already included estimates for acidic and basic components.^34^ Indeed, parameters calculated with Panayiotou's method can in principle be used in our method when experimental solvent parameters are not available.

For our data set with very different types of solutes, we improved the quality of the predictions enormously compared with Hansen's original method. Therefore, in retrospect, one might be surprised that Hansen's method worked in the first place for the paint and polymer industries. The simple reason is that for polymers the solute solubility radii are very small, utterly overshadowing the importance of the solvent radii, and thus corrections #1, #2, and #5 have essentially no effect. For small solute molecules, however, these corrections are highly important, as shown by our results. Nevertheless, there is still room for improvements. A very practical point is that Hansen's definition of the best fit is ill defined as it is based on linear errors, as already discussed in the Methods section. As a result, the fitting procedure lacks some robustness and our predictions may not yet be optimal.

Moreover, our implementation of donor and acceptor parameters is still imperfect. Many of the solutes and solvents in our data set have multiple functional groups and their parameters are now averaged into one donor and one acceptor parameter. On top of that, large molecules with one strong donating group or small molecules with a weaker donating group have the same parameter values, although their interactions with acceptor molecules may differ depending on the properties of the acceptor. Even though fitting of averaged donor and acceptor parameters was shown to be effective,^35^ more correct would be to work out all hydrogen‐bonding pairs from both molecules. But implementing that into a solubility parameter approach is not straightforward.

Another approach to solubility predictions is the work of Ruelle and Huyskens and co‐workers.^36^, ^37^ They introduced a very interesting approach to calculate entropy effects that are influenced by preferential interactions such as hydrogen bonds. Implementing their treatment of entropy into our improved Hansen methodology would probably give a further improvement. However, to do that, we would again have to let go of the averaged handling of multiple groups, so implementation into our approach is again not straightforward.

A final opportunity to further improve our methodology would be to add a temperature‐dependent Boltzmann weighing term to the polar and hydrogen‐bonding interactions, as already discussed in the Theory section. Nevertheless, with the improvements proposed in the current work we already improve significantly upon the original Hansen method, as witnessed by the results. It should be noted, though, that these improvements are based on the thermodynamics of dissolution. Other applications of Hansen parameters, such as swelling, permeability, or gel formation have different thermodynamics, so different corrections may apply.

## 5 Conclusions

The philosophy of Hansen solubility parameters is correct in using parameters for the three fundamental chemical interactions. However, the original methodology lacks thermodynamics, making it less suitable for non‐polymeric solutes. Here, we have made Hansen's method thermodynamically sound by adding simple corrections based on the entropy and enthalpy of mixing and melting.

Our improved methodology was tested on an industrial dataset of 15 solutes of very different types, for which the solubility was predicted in unseen solvents and solvent blends. Compared with Hansen's original method, the percentage of correct predictions for this dataset improved impressively from 54 % to 78 %. The most important corrections leading to this large improvement were found to be the introduction of solvent solubility radii and a correction for the “melting” of solid solutes. Also, split donor and acceptor parameters were applied, but the success of this was somewhat hampered by the necessity to average over several functional groups per molecule.

Additionally, we have shown that with the improved methodology predictions can be made at extrapolated temperatures. All in all, we have made a large step in improving Hansen's solubility parameter approach for solubility predictions of polymers as well as non‐polymeric solutes in unseen solvents and solvent blends.

## Conflict of interest

*The authors declare no conflict of interest*.

## Supporting information

As a service to our authors and readers, this journal provides supporting information supplied by the authors. Such materials are peer reviewed and may be re‐organized for online delivery, but are not copy‐edited or typeset. Technical support issues arising from supporting information (other than missing files) should be addressed to the authors.

Supplementary

## References

[1] S. Gupta , J. D. Olson , Ind. Eng. Chem. Res. 2003, 42, 6359–6374.

[2] B. C. Hancock , P. York , R. C. Rowe , Int. J. Pharm. 1997, 148, 1–21.

[3] A. F. M. Barton , Chem. Rev. 1975, 75, 731–753.

[4] J. H. A. Hildebrand , Chem. Rev. 1949, 44, 37–45. 18125399 10.1021/cr60137a003

[5] C. M. Hansen , J. Paint Technol. 1967, 39, 104–117.

[6] S. Abbott , C. M. Hansen , H. Yamamoto , Hansen Solubility Parameters in Practice, 4th ed., Hansen-Solubility.com, Denmark, 2013.

[7] C. M. Hansen , Prog. Org. Coat. 2004, 51, 77–84.

[8] T. B. Nielsen , C. M. Hansen , Polym. Test. 2005, 24, 1054–1061.

[9] A. Voelkel , B. Strzemiecka , K. Adamska , K. Milczewska , J. Chromatogr. A 2009, 1216, 1551–1566. 19010482 10.1016/j.chroma.2008.10.096

[10] T. V. M. Sreekanth , K. S. Reddy , J. Appl. Polym. Sci. 2008, 108, 1761–1769.

[11] A. Alhalaweh , A. Alzghoul , W. Kaialy , Drug Dev. Ind. Pharm. 2014, 40, 904–909. 23627441 10.3109/03639045.2013.789906

[12] A. Benazzouz , L. Moity , C. Pierlot , V. Molinier , J. Aubry , Colloids Surf. A 2014, 458, 101–109.

[13] A. J. Guenthner , K. R. Lamison , L. M. Lubin , T. S. Haddad , J. M. Mabry , Ind. Eng. Chem. Res. 2012, 51, 12282–12293.

[14] M. Raynal , L. Bouteiller , Chem. Commun. 2011, 47, 8271–8273. 10.1039/c1cc13244j 21709882

[15] J. Bonnet , G. Suissa , M. Raynal , L. Bouteiller , Soft Matter 2014, 10, 3154–3160. 24643414 10.1039/c4sm00244j

[16] J. Gao , S. Wu , M. A. Rogers , J. Mater. Chem. 2012, 22, 12651–12658.

[17] J. M. Hughes , D. Aherne , J. N. Coleman , J. Appl. Polym. Sci. 2013, 127, 4483–4491.

[18] See any physical chemistry textbook or http://en.wikipedia.org/wiki/Entropy_of_mixing.

[19] C. M. Hansen , Hansen Solubility Parameters–A User's Handbook , 2nd ed., CRC Press, Boca Raton, FL, 2007.

[20] M. H. Abraham , Chem. Soc. Rev. 1993, 22, 73–83.

[21] C. A. Hunter , Angew. Chem. Int. Ed. 2004, 43, 5310–5324; 10.1002/anie.200301739 15468180

[22] A. Beerbower , P. L. Wu , A. Martin , J. Pharm. Sci. 1984, 73, 179–188. 6707879 10.1002/jps.2600730210

[23] P. Bennema , J. van Eupen , B. M. A. van der Wolf , J. H. Los , H. Meekes , Int. J. Pharm. 2008, 351, 74–91. 17980982 10.1016/j.ijpharm.2007.09.021

[24] C. Reichardt , T. Welton , Solvents and Solvent Effects in Organic Chemistry, 4th ed., Wiley-VCH, Weinheim, 2011.

[25] F. Gharagheizi , J. Appl. Polym. Sci. 2007, 103, 31–36.

[26] G. C. Vebber , P. Pranke , C. N. Pereira , J. Appl. Polym. Sci. 2014, 131, 39696.

[27] M. Weng , J. Appl. Polym. Sci. 2016, 133, 43328.

[28] G. Rothenberg , Catal. Today 2008, 137, 2–10.

[29] D. S. Abrams , J. M. Prausnitz , AIChE J. 1975, 21, 116–128.

[30] A. Klamt , F. Eckert , Fluid Phase Equilib. 2000, 172, 43–72.

[31] G. Járvás , C. Quellet , A. Dallos , Fluid Phase Equilib. 2011, 309, 8–14.

[32] E. Stefanis , I. Tsivintzelis , C. Panayiotou , Fluid Phase Equilib. 2006, 240, 144–154.

[33] E. Stefanis , C. Panayiotou , Int. J. Thermophys. 2008, 29, 568–585.

[34] E. Stefanis , C. Panayiotou , Int. J. Pharm. 2012, 426, 29–43. 22260970 10.1016/j.ijpharm.2012.01.001

[35] E. Burello , G. Rothenberg , Adv. Synth. Catal. 2003, 345, 1334–1340.

[36] P. Ruelle , C. Rey-Mermet , M. Buchmann , H. Nam-Tran , U. W. Kesselring , P. L. Huyskens , Pharm. Res. 1991, 8, 840–850. 1656421 10.1023/a:1015891126287

[37] P. Ruelle , M. Buchmann , H. Nam-Tran , U. W. Kesselring , Pharm. Res. 1992, 9, 788–791. 1409362 10.1023/a:1015859723368

