# Supplementary material for: Revisiting Hansen Solubility Parameters by Including Thermodynamics
Source: Chemphyschem. 2017 Oct 6;18(21):2999–3006. doi: 10.1002/cphc.201700408 (PMC5725732; doi:10.1002/cphc.201700408)
Supplement: Supplementary file 1 — Supplementary [file CPHC-18-2999-s001.pdf]

CHEMPHYSCHEM

Supporting Information

Revisiting Hansen Solubility Parameters by Including Thermodynamics

Manuel J. Louwerse,[a, b] Ana Maldonado,[a, c] Simon Rousseau,[d] Chloe Moreau-Masselon,[d] Bernard Roux,[c] and Gadi Rothenberg*[a]

cphc_201700408_sm_miscellaneous_information.pdf

Supporting Information

This supporting information file contains three sections:

1.	Estimation of split Hbond parameters from data of C. A. Hunter	p1
2.	Derivation of mixing rules for solvent blends	p4
3.	Description of the optimization procedure	p7

1. Estimation of split Hbond parameters from data of C. A. Hunter[1]

In the improved Hansen parameter method, the Hbond parameter, δ_H, is now split in a donor and an acceptor parameter, δ_{HD} and δ_{HA}. In this appendix it is described how to find values for these new parameters for additional solvents.

First, let's define the various parameters:
In the original Hansen method, δ_H is the square root of the Hbond interaction per volume (of a pure substance). Hence the total Hbond interaction is: δ_H^2. If hydrogen bonds would be non-specific, it follows that the interaction between different molecules, A and B, would be: $\delta_{H,A} \cdot \delta_{H,B}$. However, since hydrogen bonds are specific interactions between donors and acceptors, we have introduced split parameters, such that the interaction per volume in a pure substance (δ_H^2) now is: $\delta_{HD} \cdot \delta_{HA}$. (Note that this is a different definition than Hansen uses in their trial to implement donor–acceptor splits; what they do there is physically incorrect, see main text). For the interaction between molecules A and B, we now obtain: $(\delta_{HD,A} \cdot \delta_{HA,B} + \delta_{HD,B} \cdot \delta_{HA,A})/2$.

While the original δ_H can be measured experimentally (albeit indirectly), the splitted δ_{HD} and δ_{HA} are not easily found experimentally, because they always come together. Fortunately, C. A. Hunter has come up with a good (i.e. correct) way to find donor and acceptor strengths from first principles.[1] Combined with experimental knowledge, he compiled a large table of values for donor and acceptor strengths for many types of molecules, see Table S1.

The α and β values of Hunter are values per mole, assuming only hydrogen bonds between the strongest donors and the strongest acceptors (one per molecule) are formed. For simple molecules with one donor and one acceptor, $\alpha \cdot \beta$ gives the total hydrogen bond interaction energy per mole. When several donor and acceptor groups are available per molecule, the $\alpha \cdot \beta$ values for each hydrogen bond that is formed should be summed.

1 [1] C. A. Hunter, *Angew. Chem. Int. Ed.* **2004**, *43*, 5310-5324.

Table S1. *Hydrogen-bond parameters for common functional groups and solvents in order of increasing hydrogen-bond strength. Copied from ref. 1.*

H-bond donors	α[a]	α[b]	H-bond donors	α[a]	α[b]	H-bond donors	α[a]	α[b]
alkane		0.4	alkyl ketone	1.5		carbamate	2.8	
alkene		0.7	amine		1.5	sulfonamide		2.8
alkyl ether		0.9	aldehyde		1.6	amide	2.9	
benzene		1.0	furan		1.7	urea	3.0	
alkyl thioether		1.0	thiol		1.7	pyrrole	3.0	
alkyl iodide		1.2	acetonitrile	1.7		sulfinamide		3.2
alkyl fluoride		1.2	thiophenol	1.8		thioamide		3.3
alkyl chloride		1.3	nitromethane	1.8		carboxylic acid	3.6	
alkyl bromide		1.3	dichloromethane	1.9		imidazole		3.7
aryl chloride		1.3	alkyne	1.9		2,2,2-trifluoroethanol	3.7	
carbon tetrachloride	1.4		1,1,2,2-tetrachloroethane		2.0	phenol	3.8	
aryl ether		1.4	ammonia		2.0	trifluoroacetic acid		3.9
aryl fluoride		1.4	aniline	2.1		phosphoric acid		4.0
pyridine		1.4	chloroform	2.2		hexafluoropropan-2-ol	4.5	
1,1,1-trichloroethane		1.5	alcohol	2.7		perfluoro-tert-butanol	4.9	
alkyl ester		1.5	water	2.8				
**H-bond acceptors**	β[a]	β[b]	**H-bond acceptors**	β[a]	β[b]	**H-bond acceptors**	β[a]	β[b]
alkane		0.3	alkyne	2.7		alcohol	5.8	
carbon tetrachloride	0.6		isothiocyanate	2.7		imine	5.8	
perfluoroalkane		0.7	thiol	2.7		ketone	5.8	
chloroform	0.8		hexafluoropropan-2-ol		3.1	sulfonamide	5.8	
dichloromethane	1.1		alkyl selenide	3.4		thioamide	5.8	
alkene	1.1		thioether	3.6		ammonia	6.1	
1,1,2,2-tetrachloroethane		1.3	nitroalkane	3.7		sulfone	6.3	
1,1,1-trichloroethane		1.4	aryl ether	3.7		pyridine	7.0	
aryl chloride	1.6		disulfide	3.7		carbamate	7.3	
aryl bromide	1.6		trifluoracetic acid		3.8	amine	7.8	
aryl iodide	1.6		pyrrole	4.1		sulfinamide	8.3	
aryl fluoride	1.6		2,2,2-trifluoroethanol		4.2	amide	8.3	
alkyl chloride	2.2		water	4.5		urea	8.3	
alkyl bromide	2.2		aldehyde	4.7		phosphinate diester	8.9	
alkyl iodide	2.2		nitrile	4.7		phosphonate diester	8.9	
benzene	2.2		sulfate diester	4.7		sulfoxide	8.9	
furan	2.2		thiocyanate	4.7		amidine	8.9	
thiophenol	2.2		carboxylic acid	5.3		phosphoric acid		9.3
perfluoro-tert-butanol		2.3	alkyl ether	5.3		phosphine oxide	9.9	
phenol	2.7		aniline	5.3				
alkyl fluoride	2.7		ester	5.3				

[a] Values based on literature values. [b] Values based on the molecular electrostatic potential surface.

To translate these α and β values to solubility parameters, a few issues need to be considered:
Most practical molecules have several donor and acceptor groups and not necessarily in equal amounts. To calculate the interaction energies in mixtures of such molecules correctly, one should keep track of all α and β values of all groups in all molecules. Then, to calculate the interaction energy for a mixture of A en B, one should sum the values of the strongest donor times the strongest acceptor in A or B, plus the second strongest donor times the second strongest acceptor, etc. (this may include AA or BB interactions.)
However, implementing this as such (with multiple α and β values per molecule) into a method for fitting solubility spheres would require too much specific knowledge about the solute molecules and would hamper the fitting procedure as well (by introducing an unknown number of variables). For the solute molecules we need to fit one donor and one acceptor parameter. Hence, for the solvent molecules we also need to translate the α and β values into one donor and one acceptor parameter, even though this is somewhat approximate.
Furthermore, the α and β values per mole need to be translated to δ_{HD} and δ_{HA} values per volume. For molecules with single donor and acceptor groups this is straightforward: one should divide by the molecular volume. However, since for most molecules the parameters are some kind of averages over the different functional groups, also the absolute values of the parameters become more uncertain. Therefore, we have chosen to calibrate the δ_{HD} and δ_{HA} parameters to the known values of the original δ_H parameter: scaling δ_{HD} and δ_{HA} until $\delta_{HD}{\cdot}\delta_{HA}$ equals δH^2.

Now let's discuss the exact method to estimate δ_{HD} and δ_{HA}:
First a list is made of all strong donors and acceptors in the molecule (ignoring CH donors at first). Then, since equal numbers of donors and acceptors are needed, weak donors or acceptors (usually only donors) are added until the correct amounts are obtained. In practice, this means that CH donors are added to the list until all acceptors are satisfied. If there are not enough weak donors or acceptors available, zeros are added in their place. Note that all oxygen atoms and double bonds are added to the list of acceptors twice, because they can accept two Hbonds each. Then, the strengths of the listed donors are summed and the same is done for the acceptors, and finally, the resulting δ_{HD} and δ_{HA} values are scaled until $\delta_{HD}\cdot\delta_{HA}$ equals δ_H^2.

Working out a few examples:
Ethanol has 1 donor: an alcohol group with a donor strength 2.7 and 1 acceptor that is counted twice: the same alcohol group with an acceptor strength of 5.8. Since the acceptor is counted twice, we need to add one CH donor: a CH group that neighbors the alcohol O, so it has the donor strength of an alkyl ether, 0.9. Now the donor strengths are summed: 2.7 + 0.9 = 3.6, and so are the acceptor strengths: 2 x 5.8 = 11.6. Finally, the numbers are scaled to fulfill the total δ_H value of 19.4: 3.6 x 11.6 = 41.76 and 19.4^2 = 376.36, so the numbers need to be scaled with $(376.36/41.76)^{1/2}$: δ_{HD} = 3.002 x 3.6 = 10.81 and δ_{HA} = 3.002 x 11.6 = 34.82.
Ethyl acetate has no strong donors and 2 acceptors: the ester bridging O (ester: 5.3) and the ester double-bonded O (ketone: 5.8). Since both acceptors are counted double, we need four donors: 3 alkyl ester CHs (neighboring the ester carbonyl) with strength 1.5 and 1 alkyl ether (neighboring the ester bridging O) with strength 0.9. Adding the donors gives: 3 x 1.5 + 0.9 = 5.4. Adding the acceptors gives: 2 x 5.3 + 2 x 5.8 = 22.2. Scaling to the known δ_H value of 7.2 gives: δ_{HD} = 0.658 x 5.4 = 3.55 and δ_{HA} = 0.658 x 22.2 = 14.60.
Formamide has two donors: two amide NH bonds with donor strength 2.9. The N-C=O moiety acts as 1 acceptor (as checked with DFT) with strength 8.3 (amide), but it still is counted double because the oxygen can accept two hydrogen bonds. No extra CH donors are needed to fill the count but otherwise the hydrogen neighboring the amide CO would simply count as an aldehyde (strength 1.6). The summed donor strength thus becomes: 2 x 2.9 = 5.8; the summed acceptor strength is: 2 x 8.3 = 16.6. Scaling to the known δ_H value of 19.0 gives: δ_{HD} = 1.936 x 5.8 = 11.23 and δ_{HA} = 1.936 x 16.6 = 32.14.

For a few molecules we have made an exception:
Chloroform has only one donor (CH with strength 2.2) and three acceptors (chlorides with strength 0.8). According to the rule above two donors with strength zero should be added: donors: 2.2 + 0 + 0 = 2.2; acceptors: 3 x 0.8 = 2.4. However, since it is known that chloroform acts much more as a donor than as an acceptor, we decided to not perform this correction in this case, so donor = 2.2 and acceptor = 0.8. Scaling to the known δ_H value of 5.7, this gives: δ_{HD} = 4.297 x 2.2= 9.45 and δ_{HA} = 4.297 x 0.8 = 3.44.
Tetrachloroethylene has no donors whatsoever and hence has a δ_{HD} of zero. This fits with the δ_H being zero. Conversely, the acceptor strength is 4 x 1.3 = 5.2. However, because of the zeros, there is no mathematical way to scale this acceptor strength to get a δ_{HA}. Hence, we copied the value of trichloroethane for this molecule: δ_{HA} = 1.9 (and δ_{HD} = 0).

For some other solvents that may be used regularly, we obtained the following parameter values:
Toluene: δ_H = 2.0; δ_{HD} = 1.35; δ_{HA} = 2.97
Diethyl ether: δ_H = 4.6; δ_{HD} = 1.90; δ_{HA} = 11.16
Acetone: δ_H = 7.0; δ_{HD} = 3.56; δ_{HA} = 13.76
Acetonitrile: δ_H = 6.1; δ_{HD} = 3.67; δ_{HA} = 10.14
Methanol: δ_H = 22.3; δ_{HD} = 12.42; δ_{HA} = 40.03
Water: δ_H = 42.3; δ_{HD} = 33.37; δ_{HA} = 53.63

2. Derivation of mixing rules for solvent blends

In the original Hansen method, the Hansen parameters for solvent blends can be calculated by a simple linear interpolation of the constituting solvents' parameters on the basis of the volume/volume ratio of the solvents in the blend. However, like the original Hansen methodology itself, this rule is only empirical, and to the best of our knowledge no mathematical proof has ever been given for this mixing rule.

Now that we improved the methodology from empirical to thermodynamically correct, we can check whether this empirical mixing rule is also thermodynamically correct. Moreover, we need to derive mixing rules for the newly introduced parameters.

Mixing rules for δ_D, δ_P, and δ_H

In the following we consider the dissolution of solute S in a blend of solvents A and B, where x' is the volume fraction of A in the mixture AB and y' is the volume fraction of S. First, let's define the enthalpy of mixing for the blend itself:

When mixing small amounts of B into A, for every volume of B that is added, all B–B interactions are broken, which costs a summed energy E_{BB}. When molecules A en B have equal sizes, the same amount of A–A interactions is broken and twice that amount A–B interactions are formed. Thus, the enthalpy of mixing of A and B is:

$$\Delta H_{AB} = (2E_{AB} - E_{AA} - E_{BB}) \qquad (1)$$

For larger amounts of B, assuming regular mixing, the volume fraction, x', comes into the equation:

$$\Delta H_{AB} = x'(1 - x')(2E_{AB} - E_{AA} - E_{BB}) \qquad (2)$$

Similarly, for the dissolution of S in pure A or pure B:

$$\Delta H_{SA} = y'(1 - y')(2E_{SA} - E_{AA} - E_{SS}) \qquad (3)$$

$$\Delta H_{SB} = y'(1 - y')(2E_{SB} - E_{BB} - E_{SS}) \qquad (4)$$

Again similarly, the enthalpy of mixing for dissolution of S in AB (still assuming regular mixing) is:

$$\Delta H_{S(AB)} = y'(1 - y')\big(2x'E_{SA} + 2(1 - x')E_{SB} - E_{SS} - (x'^2E_{AA} + (1 - x')^2E_{BB} + 2x'(1 - x')E_{AB})\big) \qquad (5)$$

In terms of ΔH_{SA} etc. that becomes:

$$\Delta H_{S(AB)} = x'\Delta H_{SA} + (1 - x')\Delta H_{SB} - y'(1 - y')\Delta H_{AB} \qquad (6)$$

If we now look at the relation between δ and ΔH (for regular mixing),

$$\Delta H_{AB} = -\beta x'(1 - x') = \tfrac{1}{2}x'(1 - x')\big[(\delta_{D,A} - \delta_{D,B})^2 + (\delta_{P,A} - \delta_{P,B})^2 + (\delta_{H,A} - \delta_{H,B})^2\big] \qquad (7)$$

, we can substitute equation 7 (and similar ones for ΔH_{SA}, ΔH_{SB}, and $\Delta H_{S(AB)}$) into equation 6, resulting in:

$$\begin{aligned} y'(1 - y')\big[(\delta_{D,S} - \delta_{D,AB})^2 + (\delta_{P,S} - \delta_{P,AB})^2 + (\delta_{H,S} - \delta_{H,AB})^2\big] \\ = x'y'(1 - y')\big[(\delta_{D,S} - \delta_{D,A})^2 + (\delta_{P,S} - \delta_{P,A})^2 + (\delta_{H,S} - \delta_{H,A})^2\big] \\ + (1 - x')y'(1 - y')\big[(\delta_{D,S} - \delta_{D,B})^2 + (\delta_{P,S} - \delta_{P,B})^2 + (\delta_{H,S} - \delta_{H,B})^2\big] \\ - y'(1 - y')x'(1 - x')\big[(\delta_{D,A} - \delta_{D,B})^2 + (\delta_{P,A} - \delta_{P,B})^2 + (\delta_{H,A} - \delta_{H,B})^2\big] \end{aligned} \qquad (8)$$

Dividing everything by $y'(1-y')$ and writing only the δ_D part of the equation, we obtain:

$$(\delta_{D,S} - \delta_{D,AB})^2 = x'(\delta_{D,S} - \delta_{D,A})^2 + (1 - x')(\delta_{D,S} - \delta_{D,B})^2 - x'(1 - x')(\delta_{D,A} - \delta_{D,B})^2 \quad (9)$$

Working out the squares and noticing the $\delta_{D,S}^2$ terms cancel, we obtain:

$$\delta_{D,AB}{}^2 - 2\delta_{D,S}\delta_{D,AB} = x'\left(\delta_{D,A}{}^2 - 2\delta_{D,S}\delta_{D,A}\right) + (1 - x')\left(\delta_{D,B}{}^2 - 2\delta_{D,S}\delta_{D,B}\right) - x'(1 - x')\left(\delta_{D,A}{}^2 + \delta_{D,B}{}^2 - 2\delta_{D,A}\delta_{D,B}\right) \quad (10)$$

Collecting terms further, we obtain:

$$\delta_{D,AB}{}^2 - 2\delta_{D,S}\delta_{D,AB} = \left[x'^2\delta_{D,A}{}^2 + (1 - x')^2\delta_{D,B}{}^2 + 2x'(1 - x')\delta_{D,A}\delta_{D,B}\right] - 2x'\delta_{D,S}\delta_{D,A} - 2(1 - x')\delta_{D,S}\delta_{D,B} \quad (11)$$

And the solution of equation 11 is:

$$\delta_{D,AB} = x'\delta_{D,A} + (1 - x')\delta_{D,B} \quad (12)$$

The same can be done for δ_P and δ_H, showing that the mixing rule for each term is indeed simple linear interpolation.

Molecules of different sizes

When molecules A, B, and S don't have equal sizes, equations 1-5 become slightly more complicated: When molecules A and B are of equal size, the summed energies E_{AA} and E_{BB} are equal to the total cohesive energies per volume in pure A and B, respectively. However, when molecules A are smaller than molecules B, E_{BB} is still the total cohesive energy, but E_{AA} is only the sum of the A–A bonds that is broken when liquid A is broken in pieces of the size of molecules B. Imagine that exactly two molecules A fit in one molecule B; replacing these two molecules A for one molecule B will break all bonds of these A molecules with other molecules A, but the interaction between the two molecules will stay. Hence, in this case E_{AA} is smaller than the total cohesive energy per volume A.

More precise: to dissolve a volume of B, the same area of A–A interactions need to be broken as the area of B–B interactions that is broken. We call this $E_{AA(B)}$, the total summed energy of A–A interactions over a molecular area of the size of the total molecular area present in a volume of pure B. Similarly, for dissolution of S in A or B, $E_{AA(S)}$ and $E_{BB(S)}$ are defined as the sum of all interactions over the total molecular area present in a volume of pure S (assuming molecules S are larger than molecules A and B).

Hence, when S > B > A, in equations 2, 3, and 4, E_{AA}, E_{AA}, and E_{BB} should be replaced by $E_{AA(B)}$, $E_{AA(S)}$, and $E_{BB(S)}$, respectively, and in equation 5 we get the terms $E_{AA(S)}$, $E_{BB(S)}$, and $E_{AB(S)}$. Now, when we collect all terms, equation 6 becomes:

$$\Delta H_{S(AB)} = x'\ \Delta H_{SA} + (1 - x')\Delta H_{SB} - y'\ (1 - y')\Delta H_{AB} + x'\ (1 - x')y'\ (1 - y')\left[E_{AA(B)} - E_{AA(S)} + E_{BB(B)} - E_{BB(S)} - 2E_{AB(B)} + 2E_{AB(S)}\right] \quad (13)$$

Thus, we see there is only a correction term when molecules S are larger than molecules B, and the correction is opposite in sign than ΔH_{AB}. Thus, when the solute has small molecules, the correct estimate of a solvent blend's δ parameters is linear interpolation, also when the solvents in the blend have different sized molecules. But when the solute has large molecules, a blend will always dissolve it slightly better than its δ parameters suggest, especially when the solvents in the blend have difficulty mixing. Note that a blend that mixes exothermally will be a somewhat *worse* solvent than its δ parameters suggest, but only when the solute has large molecules.

Mixing rules for δ_{HD} and δ_{HA}

Next we derive the mixing rules for the newly introduced δ parameters for hydrogen bond donor and acceptor strength, δ_{HD} and δ_{HA}. This means that in equation 7 $(\delta_{\text{H,A}} - \delta_{\text{H,B}})^2$ is replaced by $(\delta_{\text{HD,A}}\delta_{\text{HA,B}} + \delta_{\text{HD,B}}\delta_{\text{HA,A}} - \delta_{\text{HD,A}}\delta_{\text{HA,A}} - \delta_{\text{HD,B}}\delta_{\text{HA,B}})$, which leads to an equation for δ_{HD} and δ_{HA} similar to equation 9:

$$\begin{aligned}
\delta_{\text{HD,S}}\delta_{\text{HA,AB}} + \delta_{\text{HD,AB}}\delta_{\text{HA,S}} - \delta_{\text{HD,S}}\delta_{\text{HA,S}} - \delta_{\text{HD,AB}}\delta_{\text{HA,AB}} &= x'(\delta_{\text{HD,S}}\delta_{\text{HA,A}} + \delta_{\text{HD,A}}\delta_{\text{HA,S}} - \delta_{\text{HD,S}}\delta_{\text{HA,S}} - \delta_{\text{HD,A}}\delta_{\text{HA,A}}) \\
&+ (1 - x')(\delta_{\text{HD,S}}\delta_{\text{HA,B}} + \delta_{\text{HD,B}}\delta_{\text{HA,S}} - \delta_{\text{HD,S}}\delta_{\text{HA,S}} - \delta_{\text{HD,B}}\delta_{\text{HA,B}}) \\
&- x'(1 - x')(\delta_{\text{HD,A}}\delta_{\text{HA,B}} + \delta_{\text{HD,B}}\delta_{\text{HA,A}} - \delta_{\text{HD,A}}\delta_{\text{HA,A}} - \delta_{\text{HD,B}}\delta_{\text{HA,B}})
\end{aligned} \tag{14}$$

Working out some brackets and collecting all terms, we obtain:

$$\begin{aligned}
\delta_{\text{HD,S}}\delta_{\text{HA,AB}} + \delta_{\text{HD,AB}}\delta_{\text{HA,S}} - \delta_{\text{HD,AB}}\delta_{\text{HA,AB}} &= [x'\delta_{\text{HD,S}}\delta_{\text{HA,A}} + (1 - x')\delta_{\text{HD,S}}\delta_{\text{HA,B}}] + [x'\delta_{\text{HD,A}}\delta_{\text{HA,S}} + (1 - x')\delta_{\text{HD,B}}\delta_{\text{HA,S}}] \\
&- [x'^2\delta_{\text{HD,A}}\delta_{\text{HA,A}} + (1 - x')^2\delta_{\text{HD,B}}\delta_{\text{HA,B}} + x'(1 - x')\delta_{\text{HD,A}}\delta_{\text{HA,B}} \\
&+ x'(1 - x')\delta_{\text{HD,B}}\delta_{\text{HA,A}}]
\end{aligned} \tag{15}$$

With again as obvious solution:

$$\delta_{\text{HA,AB}} = x'\delta_{\text{HA,A}} + (1 - x')\delta_{\text{HA,B}} \quad \text{and}$$
$$\delta_{\text{HD,AB}} = x'\delta_{\text{HD,A}} + (1 - x')\delta_{\text{HD,B}} \tag{16}$$

Hence we see that also for δ_{HD} and δ_{HA} the correct mixing rule is linear interpolation on the basis of the volume/volume ratio of the solvents in the blend.

Mixing rule for $r_{solvent}$

Finally, we have introduced a solubility radius for the solvent, so in the case of solvent blends we need a mixing rule for this r_{solvent}, as well. In our work we use the assumption that r_{solvent} is inversely proportional to the molar volume. The mixing rule derived here follows from the same assumption. Obviously when non-ideal behavior of some solvents is taken into account, leading to different solvent radii, also the mixing rule may deviate.

Another way of saying that r_{solvent} is inversely proportional to the molar volume is to say it is proportional to the number of molecules per liter. The number of molecules in a mixture of A en B, where x' is the volume fraction of A in the blend, is:

$$N = \left(\frac{x'}{V_{\text{M,A}}} + \frac{1-x'}{V_{\text{M,B}}}\right)V \tag{17}$$

This straightforwardly leads to:

$$r_{\text{AB}} = x'r_{\text{A}} + (1 - x')r_{\text{B}} \tag{18}$$

Hence we see that for all parameters conveniently the same mixing rule applies.

3. Description of the optimization procedure

Since optimizing the best fit for (improved) Hansen spheres is not straightforward, here we describe our procedure as implemented in the script made available. In fact, this procedure is not perfect, as touched upon in the main text, but in combination with using a grid of starting points it does the job.

First of all, a starting estimate is made by averaging the parameters of all solvents that do dissolve the solute at hand. Then a grid of 129 starting positions is set up with a maximum distance of four units from that first estimate. For each of these starting positions first the sphere radius is optimized followed by an optimization of all other parameters, as detailed below, until convergence is reached. Finally out of the 129 optimizations, the best one is selected and given as output.

At each step of the optimization, for each solvent the parameter distance to the current center of the Hansen sphere is calculated taking into account all described improvements, and the minimum radius for the sphere is calculated to include the data point (taking into account the inverse sum of radii). Then derivatives are calculated to find the direction to improve the fitted parameters. It should be noted, though, that these derivatives are discontinuous when the data points enter or leave the fitted sphere (whenever a point is at the correct side of the sphere, the derivatives fall to zero immediately), making convergence somewhat cumbersome.
After every optimization step of the other parameters the sphere radius is fully re-optimized. The donor/acceptor parameters, δ_{HD} and δ_{HA}, are optimized alongside with the main Hansen parameters, while at every step they are rescaled to be consistent with the δ_H parameter. For the ratio between δ_{HD} and δ_{HA} a maximum ratio of 20 was used. The c_{melt} parameter (c_solid in the code) is optimized while optimizing the radius.

When a perfect fit is found, the optimization for that starting point is stopped. In that case, at the end, all perfect solutions are averaged and –after re-optimizing the radius– the average is given as output.

Note that when a narrow perfect fit or an almost perfect fit of the data exists, the implemented procedure has difficulty in converging to the best fit, probably due to the Boolean nature of the data. Adding a small randomness may help to overcome these difficulties. For that reason at some points in the code we have deviated slightly from the mathematically correct derivatives when this was found to help convergence.

All in all, the optimization procedure can likely be improved by combining it with a method to search for possible perfect fits first, but due to all the non-linear improvements introduced in our work this is mathematically challenging. Nonetheless, by starting the optimization from a grid of starting points, the current procedure gives good results.

input_fitresults

fitresults

#name deltaD deltaP deltaH donor acceptor radius melting_correction molar_volume melting_point

Solute1 18.0925 7.55555 6.53719 6.01725 7.04154 4.66913 6.8 256 432 #Based on high and low temperature data

Solute2 17.2525 11.3424 10.5254 7.80387 14.1944 4.55527 5.7 237 474 #Based on room temperature only

input_options

options

#lines starting with a "#" are ignored.

useorigHansen 0 # 0 or 1 (origHansen should be combined with xfactor=2, but this is not enforced)

xfactor 1 # 1 or 2

correct4solid 1 # 0 or 1; should be 1 for solids and 0 for liquids

correctc_solidfortemp 2 # 0, 1 or 2; 0 means no correction (incorrect!); 1 means ignoring heat capacity effects; 2 means average of 0 and 1 because 1 seems to overcorrect sometimes; N.B. this option only has significant effect when mixing data at different temperatures or making predictions at extrapolated temperatures (otherwise numerical effects may occur).

input_predictions

predictions

solute solvent/mix concentration(in_mol/L) yes/no/? temperature_in_K(default=295)

in case of mixtures use: "mix" nr_solvents name_solvent1 amount_solvent1 name_solvent2 amount_solvent2 etc (the sum of the amounts should be normalized to 1)

Solute1 51 1.25 ? 277

Solute1 52 1.25 ? 277

Solute1 53 1.25 ? 277

Solute1 mix 2 30 0.5 31 0.5 1.25 ? 277

Solute1 mix 2 30 0.4 31 0.6 1.25 ? 277

Solute1 mix 2 30 0.3 31 0.7 1.25 ? 277

Solute1 mix 3 21 0.3792 34 0.0987 38 0.5221 1.25 ?

Solute1 mix 3 38 0.0681 34 0.4858 5 0.4461 1.25 ?

Solute2 49 0.072 ? 273

Solute2 50 0.072 ? 273

Solute2 mix 2 43 0.7 32 0.3 0.072 ? 273

Solute2 mix 2 21 0.4 29 0.6 0.072 ? 273

Solute2 mix 2 43 0.7 32 0.3 0.072 ?

Solute2 mix 2 21 0.4 29 0.6 0.072 ?

Solute2 mix 2 21 0.6 29 0.4 0.072 ?

Solute3 A1 0.16 no 273

Solute3 A5 0.16 no 273

Solute3 formic_acid 0.16 no 273

Solute3 mix 2 25 0.5 35 0.5 0.16 yes 273

Solute3 mix 2 31 0.7 30 0.3 0.16 yes 273

Solute3 mix 2 35 0.5 23 0.5 0.16 no 273

input_solvents

solventdata

#nr_or_name description deltaD deltaP deltaH donor acceptor molar_volume

1 toluene 18 1.4 2 1.3478 2.9678 106.6

2 chloroform 17.8 3.1 5.7 9.4486 3.4386 80.5

3 1,1,1-trichloroethane 16.8 4.3 2 2.07122 1.93122 99.3

4 tetrachloroethylene 18.3 5.7 0 0 1.9 102.8

5 diethyl_ether 14.5 2.9 4.6 1.89663 11.1566 104.7

6 acetone 15.5 10.4 7 3.56083 13.7608 73.8

7 ethyl_acetate 15.8 5.3 7.2 3.55055 14.6006 98.6

8 acetonitrile 15.3 18 6.1 3.66972 10.1397 52.9

9 formamide 17.2 26.2 19 11.2316 32.1416 39.9

10 methanol 14.7 12.3 22.3 12.4222 40.0322 40.6

11 ethanol 15.8 8.8 19.4 10.809 34.819 58.6

12 water 15.5 16 42.3 33.3661 53.6261 18

formic_acid formic_acid 14.6 10 14 9.37 20.91 37.7

input_testdata

testdata

First a line must be given with the name of the compound followed by its molar volume and its melting point in Kelvin, and optionally a default temperature in Kelvin

(The molar volume can be calculated by dividing the molar mass by the density)

(For low melting points, the melting point should be measured correctly; for high melting points, any estimate should suffice)

(If no temperature is specified, room temperature (295K) is taken as default temperature)

The other lines must follow the following format (if no temperature is specified, the abovementioned default temperature is assumed):

name(or_nr)_solvent dissolves(yes/no) concentration(in_mol/L) temperature(in_K)

solute Solute1 246 387 297

1 no 0.072

mix 2 1 0.5 2 0.5 no 0.072

2 no 0.072

3 no 0.072

4 yes 0.072

5 no 0.072

6 yes 0.072

7 yes 0.072

8 yes 0.072

9 no 0.072

10 yes 0.072

1 no 0.072 273

2 no 0.072 273

3 no 0.072 273

4 no 0.072 273

5 no 0.072 273

6 yes 0.072 273

7 yes 0.072 273

8 no 0.072 273

solute Solute2 277 372

1 no 1.25

2 no 1.25

3 no 1.25

4 no 1.25

5 no 1.25

6 yes 1.25

7 yes 1.25

8 no 1.25

9 no 1.25

10 no 1.25

5 no 1.25 277

6 no 1.25 277

7 no 1.25 277

8 no 1.25 277

Manual for the Matlab function *fit_sphere*, implementing the improved Hansen methodology

Fit_sphere is the Matlab function that implements the improved Hansen methodology, written by Manuel J. Louwerse from the University of Amsterdam. The function uses several plain text inputfiles which can be invoked in random order. For each inputfile type, multiple files are allowed; all information will simply be added. There are five types of inputfiles: options, solventdata, testdata, fitresults, and predictions. The type of the file needs to be given on the first line of each inputfile. For all file types, empty lines and lines starting with a dash (#) are ignored. Also at the end of lines it is always allowed to add comments starting with a dash. Together with the Matlab function, we distribute example files of each type, containing comments describing the format. For further explanations we refer to the publication in ChemPhysChem.

The *fit_sphere* function is issued with the following command in Matlab, although different names for the inputfiles can be used:

```
fit_sphere({'input_options.txt';'input_solvents.txt';'input_testdata.txt';'inp
ut_fitresults.txt';'input_predictions.txt'})
```

The output is always written in the file "output.txt" in the working directory.

- It is required to have an options inputfile, in which several runtime options are specified, see the example file.

- Also a solventdata inputfile is required, in which the Hansen parameters, donor-acceptor splits for the deltaH parameter, and the molar volumes of all solvents are given. Note that in the names of the solvents no spaces are allowed.
  Mixtures of solvents are defined in the files where they are used: testdata or predictions inputfiles.

- One or more testdata files or fitresults files are required. Testdata files contain the experimentally determined results on which the sphere fitting is based. This file can contain data for more than 1 solute; if so, for each solute a sphere will be fitted. If, for a solute, data is given at multiple concentrations or temperatures, this will be used together, i.e. fitting one sphere with mixed data. Concentrations (in mol/L) are mandatory. Temperatures can be left out; in that case 295K is assumed.
  Alternatively, a fitresults file can be specified. The idea of this file is that it prevents having to run the slow fitting procedure over and over again when one only wants to do some quick predictions. The fitresults file should contain the results of previously performed fits. It is possible to specify both testdata files and fitresults files. In that case, the testdata will be used to fit spheres for the solutes in the testdata files and for the other solutes the data from

the fitresults files will be used. If the same solute is specified in both type of files, the old fitresults will be overruled by the new testdata.

- The predictions inputfile is not required. If present, it contains definitions of solutes and solvents for which a prediction is desired at the given concentration and temperature. Again, if no temperature is specified, 295K is assumed. If the experiment has already been done, the result (yes or no) can be given as well. The script will then keep count of the number of correct and incorrect predictions and write these counts in the output file. If the experimental result is not known, a question mark (?) should be specified.

In the testdata and predictions inputfiles, also mixtures of solvents can be defined. In that case, instead of a single column with the name or the number of the solvent, a complete definition is given of the mixture, starting with the word "mix". The format of this definition is as follows: "mix" (nr of solvents) name1 fraction1 name2 fraction2 etc.

Examples: "mix 2 10 0.5 25 0.5" means a mixture of two solvents, namely 50% of solvent nr 10 and 50% of solvent nr 25.

"mix 3 41 0.1 A10 0.7 formic_acid 0.2" means a mixture of three solvents, namely 10% of solvent 41, 70% of solvent A10, and 20% of formic_acid.

Note that the solvent fraction should always add up to 1; otherwise an error message is issued. The solvent fractions are defined as volume fractions.

Units:

The Hansen parameters D, P, H, Hdonor, and Hacceptor should be given in SI units (MPa^1/2), the same unit as typically used in the Hansen software. Temperatures and melting points are always in Kelvin. Molar volumes are expected in mL/mol, and can be calculated by dividing the molar mass (g/mol) by the density (g/mL) of the pure substance. Concentrations are given in mols solute per liter of solvent (not per liter of solution!). Solvent fractions in mixed solvents are expected in volume fractions.

Compared to the original Hansen method, two additional quantities of the solute have to be entered: the molar volume and the melting point. When measuring these quantities is problematic, an estimate of the molar volume based on a density of 1 should suffice. For polymers any large number (e.g. 1E10) can be entered for the molar volume, as long as it is consistent with the entered concentrations (e.g. 1E-11).

The melting point is only important when data with mixed temperatures is used or when predictions at extrapolated temperatures are desired. Otherwise any reasonable number suffices. Also when extrapolations are desired, the rule is that for low melting points a higher acuracy is needed than for high melting points (for

a melting point around 350K the inaccuracy should be less than 10 degrees; for a melting point around 400K, an inaccuracy of 20 degrees is still oke).

Sphere radii are in principle in the same unit as the Hansen parameters, but because in this improved method the relations are not linear anymore, one should be very careful with interpreting the sphere radius. Also it should be mentioned that the sphere radius as given by the software is extrapolated to a 1:1 molar mixing ratio between solute and solvent (note that, as a result, the software may report a negative radius; this is not a bug). The reason for this is that the software can handle mixed data for multiple concentrations and these datapoints have to be extrapolated to one single concentration in order to compare them correctly. The reason again for this is that for some equations the volume fraction matters and for other equations the molar fraction matters. As it is impossible to keep both constant, the software needs to be able to work with mixed data. As a bonus this means that this improved method can also make predictions at extrapolated concentrations, but obviously the accuracy is best when extrapolation is kept limited.

The same is true for temperatures: The software can work with mixed data and the reported sphere radius is for solubility at room temperature (295K). The Hansen parameters are considered to be the same at all temperatures.

Also, the sphere radius depends on the correction for solid melting, so it is not allowed to fiddle with the value of this correction and keep using the same value for the radius.

Finally, we stress that the scale of the solubility parameters (and especially the radius) depend on the options set in the options inputfile. Therefore, one should always make sure that while making predictions the exact same options are set as when making the fitresults file. When the options during predicting are not consistent with the options used for the fitting, the predictions will be highly incorrect.

fit_sphere

```
% Written by Manuel J. Louwerse from the University of Amsterdam

% Version 1.0  23-09-2014


function fit_sphere(filenames)  %filenames is a cell array with names of inputfiles, e.g.: fit_sphere({'input_options.txt';'input_solvents.txt'})

  global useorigHansen xfactor correct4solid correctc_solidfortemp

  maxratioDA=20;



  options_present=0; solventdata_present=0; testdata_present=0; predictions_present=0; fitresults_present=0;

  nsolvents=0; nsolutes=0; npredictions=0; nprefits=0; nmixes=0; nsolventstotal=0;

  solventx=[]; solventy=[]; solventz=[]; solventzD=[]; solventzA=[]; molvolume=[]; solventr=[];

  name_solute=cell(1); molvolume_solute=[]; Tm_solute=[];

  npuresinmix=[]; namepureinmix=cell(1,1); amountpureinmix=[];

  npredictions=0; predictsolvent=cell(1); predictsolute=cell(1); predictresult=cell(1); predictconc=[]; predicttemp=[];

  nprefits=0; prefitsolute=cell(1); prefitx=[]; prefity=[]; prefitz=[]; prefitzD=[]; prefitzA=[]; prefitr=[]; prefitc_solid=[]; prefitvolume=[]; prefitTm=[];

  npositives=[]; positive=[]; xpositive=[]; ypositive=[]; zpositive=[]; zDpositive=[]; zApositive=[]; rpositive=[]; volpositive=[]; concpositive=[]; temppositive=[];

  nnegatives=[]; negative=[]; xnegative=[]; ynegative=[]; znegative=[]; zDnegative=[]; zAnegative=[]; rnegative=[]; volnegative=[]; concnegative=[]; tempnegative=[];

  xsphere=0; ysphere=0; zsphere=0; zDsphere=0; zAsphere=0; radius=0; c_solid=0;

  closestminus=0; furthestplus=0; nextminusin=0; nextminusout=0; nextplusin=0; nextplusout=0; deltaoff=0; improvex=0; improvey=0; improvez=0; improveDonor=0; improveAcceptor=0; summinus_drdcsolid=0; sumplus_drdcsolid=0;

  offtotal=0; nminus=0; nplus=0; fitquality=0;

  nperfect=0; avgx=0; avgy=0; avgz=0; avgzD=0; avgzA=0; avgr=0; avgcsolid=0; minx=0; miny=0; minz=0; minzD=0; minzA=0; minr=0; mincsolid=0; maxx=0; maxy=0; maxz=0; maxzD=0; maxzA=0; maxr=0; maxcsolid=0;
```

```
function readinputs    %nested function within fit_sphere
  % First set some defaults:
  useorigHansen=0;
  xfactor=1;
  correct4solid=0;
  correctc_solidfortemp=2;

  % Then read in all inputfiles in random order:
  nfilenames=size(filenames,1);
  for ifile=1:nfilenames
    filenamechar=char(filenames(ifile,:));
    currentfile=fopen(filenamechar);
    filename=filenames(ifile,:);
    fileended=0;
    iline=0;
    while (fileended==0)
      linestring=fgetl(currentfile);
      if (linestring==-1)
        fclose(currentfile);
        break
      end
      iline=iline+1;
      inputdata=strsplit(linestring);

      if (iline==1)
        if (strcmp(inputdata(1),'options'))
          options_present=1;
          current=inputdata(1);
        elseif (strcmp(inputdata(1),'solventdata'))
          solventdata_present=1;
          current=inputdata(1);
```

```
      elseif (strcmp(inputdata(1),'testdata'))
        testdata_present=1;
        current=inputdata(1);
      elseif (strcmp(inputdata(1),'predictions'))
        predictions_present=1;
        current=inputdata(1);
      elseif (strcmp(inputdata(1),'fitresults'))
        fitresults_present=1;
        current=inputdata(1);
      else
        error(['Error: First line of file ',char(filename),' should be:  "options", "solventdata", "testdata", "fitresults", or "predictions". Exit.']);
      end
    end
    if (iline>1 && not(strncmp(inputdata(1),'#',1)) && not(strcmp(inputdata(1),'')))
      if (strcmp(current,'options'))
        if (strcmp(inputdata(1),'useorigHansen'))
          useorigHansen=str2double(char(inputdata(2)));
        end
        if (strcmp(inputdata(1),'xfactor'))
          xfactor=str2double(char(inputdata(2)));
        end
        if (strcmp(inputdata(1),'correct4solid'))
          correct4solid=str2double(char(inputdata(2)));
        end
        if (strcmp(inputdata(1),'correctc_solidfortemp'))
          correctc_solidfortemp=str2double(char(inputdata(2)));
        end
      end

      if (strcmp(current,'solventdata'))
```

```
      % check for duplicate information; if the solvent already has been read in, no new entry will be made but its data will be overwritten
      already=0;
      for i=1:nsolvents
        if (strcmp(name_solvent(i),inputdata(1)))
          already=1;
          solvent=i;
        end
      end
      if (already==0)
        nsolvents=nsolvents+1;
        name_solvent(nsolvents)=inputdata(1);
        solvent=nsolvents;
      end
      solventx(solvent)=str2double(char(inputdata(3)));
      solventy(solvent)=str2double(char(inputdata(4)));
      solventz(solvent)=str2double(char(inputdata(5)));
      solventzD(solvent)=str2double(char(inputdata(6)));
      solventzA(solvent)=str2double(char(inputdata(7)));
      molvolume(solvent)=str2double(char(inputdata(8)));
    end

    if (strcmp(current,'testdata'))
      if (strcmp(inputdata(1),'solute'))
        already=0;
        for i=1:nsolutes
          if (strcmp(name_solute(i),inputdata(2)))
            already=1;
            solute=i;
          end
        end
```

```
    if (already==0)
      nsolutes=nsolutes+1;
      name_solute(nsolutes)=inputdata(2);
      solute=nsolutes;
      ntestdata(solute)=0;
    end
    molvolume_solute(solute)=str2double(char(inputdata(3)));
    Tm_solute(solute)=str2double(char(inputdata(4)));
    if (size(inputdata,2)>4 && not(strncmp(inputdata(5),'#',1)))
      defaultT=str2double(char(inputdata(5)));
    else
      defaultT=295;
    end
  else
    if (not(strcmp(inputdata(1),'mix')))
      if (size(inputdata,2)>3 && not(strncmp(inputdata(4),'#',1)))
        temperature=str2double(char(inputdata(4)));
      else
        temperature=defaultT;
      end
      already=0;
      for i=1:ntestdata(solute)
        if (strcmp(testsolvent(solute,i),inputdata(1)) &&
strcmp(testresult(solute,i),inputdata(2)) &&
testconc(solute,i)==str2double(char(inputdata(3))) && testtemp(solute,i)==temperature)
          % duplicate data ignored
          already=1;
        end
      end
      if (already==0)
        ntestdata(solute)=ntestdata(solute)+1;
        testsolvent(solute,ntestdata(solute))=inputdata(1);
```

```
          testresult(solute,ntestdata(solute))=inputdata(2);

          testconc(solute,ntestdata(solute))=str2double(char(inputdata(3)));

          testtemp(solute,ntestdata(solute))=temperature;

          testfile(solute,ntestdata(solute))=filename;

          testline(solute,ntestdata(solute))=iline;

        end

      end

      if (strcmp(inputdata(1),'mix'))

        npures=str2double(char(inputdata(2)));

        if (size(inputdata,2)>4+2*npures &&
not(strncmp(inputdata((5+2*npures)),'#',1)))

          temperature=str2double(char(inputdata((5+2*npures))));

        else

          temperature=defaultT;

        end

        nsame=0;

        already=0;

        for i=1:nmixes

          if (npuresinmix(i)==npures)

            almostsame=1;

            for j=1:npures

              if (not(strcmp(namepureinmix(i,j),inputdata((1+2*j)))) ||
amountpureinmix(i,j)~=str2double(char(inputdata((2+2*j)))))

                almostsame=0;

              end

            end

            if (almostsame==1)

              nsame=nsame+1;

              same{nsame}=['mix_',num2str(i)];

            end

          end

        end
```

```
        for i=1:nsame
          for j=1:ntestdata(solute)
            if (strcmp(testsolvent(solute,j),same(i)) &&
strcmp(testresult(solute,j),inputdata((3+2*npures))) &&
testconc(solute,j)==str2double(char(inputdata((4+2*npures)))) &&
testtemp(solute,j)==temperature)
              % duplicate data ignored
              already=1;
            end
          end
        end
        if (already==0)
          nmixes=nmixes+1;
          npuresinmix(nmixes)=str2double(char(inputdata(2)));
          mixfile(nmixes)=filename;
          mixline(nmixes)=iline;
          sum=0;
          for i=1:npuresinmix(nmixes)
            namepureinmix(nmixes,i)=inputdata((1+2*i));
            amountpureinmix(nmixes,i)=str2double(char(inputdata((2+2*i))));
            sum=sum+amountpureinmix(nmixes,i);
          end
          if (sum~=1)
            error(['Error: ',char(filename),': The amounts in the mixture in line
',num2str(iline),' do not add up to 1. Exit.']);
          end
          ntestdata(solute)=ntestdata(solute)+1;
          testsolvent{solute,ntestdata(solute)}=['mix_',num2str(nmixes)];
          testresult(solute,ntestdata(solute))=inputdata((3+2*npures));

testconc(solute,ntestdata(solute))=str2double(char(inputdata((4+2*npures))));
          testtemp(solute,ntestdata(solute))=temperature;
          testfile(solute,ntestdata(solute))=filename;
```

```
        testline(solute,ntestdata(solute))=iline;
      end
    end
  end
end

if (strcmp(current,'predictions'))
  npredictions=npredictions+1;
  predictsolute(npredictions)=inputdata(1);
  if (not(strcmp(inputdata(2),'mix')))
    predictsolvent(npredictions)=inputdata(2);
    predictconc(npredictions)=str2double(char(inputdata(3)));
    predictresult(npredictions)=inputdata(4);
    if (size(inputdata,2)>4 && not(strncmp(inputdata(5),'#',1)))
      predicttemp(npredictions)=str2double(char(inputdata(5)));
    else
      predicttemp(npredictions)=295;
    end
  end
  if (strcmp(inputdata(2),'mix'))
    nmixes=nmixes+1;
    predictsolvent{npredictions}=['mix_',num2str(nmixes)];
    npuresinmix(nmixes)=str2double(char(inputdata(3)));
    mixfile(nmixes)=filename;
    mixline(nmixes)=iline;
    sum=0;
    for i=1:npuresinmix(nmixes)
      namepureinmix(nmixes,i)=inputdata((2+2*i));
      amountpureinmix(nmixes,i)=str2double(char(inputdata((3+2*i))));
      sum=sum+amountpureinmix(nmixes,i);
    end
```

```
            if (sum~=1)
              error(['Error: ',char(filename),': The amounts in the mixture in line
',num2str(iline),' do not add up to 1. Exit.']);
            end

predictconc(npredictions)=str2double(char(inputdata((4+2*npuresinmix(nmixes)))));
            predictresult(npredictions)=inputdata((5+2*npuresinmix(nmixes)));
            if (size(inputdata,2)>5+2*npuresinmix(nmixes) &&
not(strncmp(inputdata((6+2*npuresinmix(nmixes))),'#',1)))

predicttemp(npredictions)=str2double(char(inputdata((6+2*npuresinmix(nmixes)))));
            else
              predicttemp(npredictions)=295;
            end
          end
        end

        if (strcmp(current,'fitresults'))
          nprefits=nprefits+1;
          prefitsolute(nprefits)=inputdata(1);
          prefitx(nprefits)=str2double(char(inputdata(2)));
          prefity(nprefits)=str2double(char(inputdata(3)));
          prefitz(nprefits)=str2double(char(inputdata(4)));
          prefitzD(nprefits)=str2double(char(inputdata(5)));
          prefitzA(nprefits)=str2double(char(inputdata(6)));
          prefitr(nprefits)=str2double(char(inputdata(7)));
          prefitc_solid(nprefits)=str2double(char(inputdata(8)));
          prefitvolume(nprefits)=str2double(char(inputdata(9)));
          prefitTm(nprefits)=str2double(char(inputdata(10)));
        end
      end
    end
  end
```

```
    if (options_present~=1)
      error( 'Error: No options file specified.')
    end
    if (solventdata_present~=1)
      error( 'Error: No solventdata file specified.')
    end
    if (testdata_present~=1 && fitresults_present~=1)
      error( 'Error: No experimental data file and no fitresults file specified.')
    end
    if (xfactor~=1 && xfactor~=2)
      error( 'Error: Option xfactor can only be 1 or 2; otherwise the sphere radii of the
solvents are not calibrated. Exit.')
    end

  % Calibrate the sphere radii of the solvents:
    for i=1:nsolvents
      if (xfactor==1)
        solventr(i)=1520/molvolume(i);
      end
      if (xfactor==2)
        solventr(i)=1545/molvolume(i);
      end
    end

  % For all mixes the solvent parameters, the sphere radius, and the effective molecular
volumes are now calculated:
    nsolventstotal=nsolvents+nmixes;
    for i=1:nmixes
      name_solvent{nsolvents+i}=['mix_',num2str(i)];
      solventx(nsolvents+i)=0; solventy(nsolvents+i)=0; solventz(nsolvents+i)=0;
      solventzD(nsolvents+i)=0; solventzA(nsolvents+i)=0; solventr(nsolvents+i)=0;
```

```
    molvolume(nsolvents+i)=0;
    flag=0;
    for j=1:npuresinmix(i)
      exists=0;
      for k=1:nsolvents
        if (strcmp(namepureinmix(i,j),name_solvent(k)))
          ipureinmix(i,j)=k;
          exists=1;
        end
      end
      if (exists==0)
        disp(['Warning: For mix_',num2str(i),' (',char(mixfile(i)),', line
',num2str(mixline(i)),') no data available for solvent ',char(namepureinmix(i,j)),'. This
mixture will be ignored.'])
        flag=1;
      end
      if (flag==0)

solventx(nsolvents+i)=solventx(nsolvents+i)+amountpureinmix(i,j)*solventx(ipureinmix(i,
j));

solventy(nsolvents+i)=solventy(nsolvents+i)+amountpureinmix(i,j)*solventy(ipureinmix(i,
j));

solventz(nsolvents+i)=solventz(nsolvents+i)+amountpureinmix(i,j)*solventz(ipureinmix(i,
j));

solventzD(nsolvents+i)=solventzD(nsolvents+i)+amountpureinmix(i,j)*solventzD(ipurein
mix(i,j));

solventzA(nsolvents+i)=solventzA(nsolvents+i)+amountpureinmix(i,j)*solventzA(ipureinm
ix(i,j));

solventr(nsolvents+i)=solventr(nsolvents+i)+amountpureinmix(i,j)*solventr(ipureinmix(i,j
));

molvolume(nsolvents+i)=molvolume(nsolvents+i)+amountpureinmix(i,j)/molvolume(ipure
inmix(i,j))  ; %outside the loop we will do 1/x
```

```
    end
  end
  if (flag==0)
    molvolume(nsolvents+i)=1/molvolume(nsolvents+i);
    if (useorigHansen==0)
      solventz(nsolvents+i)=(solventzD(nsolvents+i)*solventzA(nsolvents+i))^0.5;
    end
  end
end

% All testdata combined with solvent parameters are now put in organised variables
for solute=1:nsolutes
  npositives(solute)=0;
  nnegatives(solute)=0;
  for j=1:ntestdata(solute)
    exists=0;
    for k=1:nsolventstotal
      if (strcmp(testsolvent(solute,j),name_solvent(k)))
        solvent=k;
        exists=1;
      end
    end
    if (exists==1)
      if (strcmp(testresult(solute,j),'yes'))
        npositives(solute)=npositives(solute)+1;
        positive(solute,npositives(solute))=solvent;
        xpositive(solute,npositives(solute))=solventx(solvent);
        ypositive(solute,npositives(solute))=solventy(solvent);
        zpositive(solute,npositives(solute))=solventz(solvent);
        zDpositive(solute,npositives(solute))=solventzD(solvent);
        zApositive(solute,npositives(solute))=solventzA(solvent);
```

```
            rpositive(solute,npositives(solute))=solventr(solvent);
            volpositive(solute,npositives(solute))=molvolume(solvent);
            concpositive(solute,npositives(solute))=testconc(solute,j);
            temppositive(solute,npositives(solute))=testtemp(solute,j);
          end
          if (strcmp(testresult(solute,j),'no'))
            nnegatives(solute)=nnegatives(solute)+1;
            negative(solute,nnegatives(solute))=solvent;
            xnegative(solute,nnegatives(solute))=solventx(solvent);
            ynegative(solute,nnegatives(solute))=solventy(solvent);
            znegative(solute,nnegatives(solute))=solventz(solvent);
            zDnegative(solute,nnegatives(solute))=solventzD(solvent);
            zAnegative(solute,nnegatives(solute))=solventzA(solvent);
            rnegative(solute,nnegatives(solute))=solventr(solvent);
            volnegative(solute,nnegatives(solute))=molvolume(solvent);
            concnegative(solute,nnegatives(solute))=testconc(solute,j);
            tempnegative(solute,nnegatives(solute))=testtemp(solute,j);
          end
        else
          disp(['Warning: No data available for solvent ',char(testsolvent(solute,j)),'. The testresult of solute ',char(name_solute(solute)),' with this solvent (',char(testfile(solute,j)),', line ',num2str(testline(solute,j)),') is ignored in the fitting.']);
        end
      end
    end
  end %end of function readinputs


  function find_sphere    %nested function within fit_sphere
    function find_startsphere    %nested function within find_sphere
      % First do a very rough estimation by simply averaging positives. (with a little trick for the zDA values)
      xsphere=0; ysphere=0; zsphere=0;
```

```
    for i=1:npositives(solute)
      xsphere=xsphere+xpositive(solute,i)/npositives(solute);
      ysphere=ysphere+ypositive(solute,i)/npositives(solute);
      zsphere=zsphere+zpositive(solute,i)/npositives(solute);
      zDsphere=zDsphere+zApositive(solute,i)/npositives(solute);
      zAsphere=zAsphere+zDpositive(solute,i)/npositives(solute);
    end
    if (useorigHansen==0)
      [zDsphere,zAsphere]=make_consistent_zDA(zsphere,zDsphere,zAsphere);
    end
    zDsphere=zsphere; %this is the old situation and somehow it seems to perform
slightly better...
    zAsphere=zsphere;
    radius=-1000;
    for i=1:npositives(solute)

[r,distx,disty,distz,realr]=parameter_distance(xsphere,ysphere,zsphere,zDsphere,zAspher
e,xpositive(solute,i),ypositive(solute,i),zpositive(solute,i),zDpositive(solute,i),zApositive(so
lute,i),molvolume(positive(solute,i)),molvolume_solute(solute));
      if (r>radius)
        radius=r;
      end
    end
  end %end of function find_startsphere


  function improve_Donor_Acceptor    %nested function within find_sphere
    if (zsphere>0)
      measure_error_symmetricradii;
      maxratio=6;
      ratio=improveDonor/improveAcceptor;
      if (improveDonor<0 && improveAcceptor<0)
        ratio=1/ratio;
      elseif (improveDonor<=0)
```

```
  ratio=1/maxratio;
elseif (improveAcceptor<=0)
  ratio=maxratio;
end
if (improveDonor==0 && improveAcceptor==0)
  ratio=1;
end
if (ratio>maxratio)
  ratio=maxratio;
end
if (ratio<1/maxratio)
  ratio=1/maxratio;
end
if (ratio~=1)
  power=offtotal/15;
  if (power>0.10)
    power=0.10;
  end
  zDsphere=zDsphere*ratio^power;
  zAsphere=zAsphere/ratio^power;
  if (zDsphere/zAsphere>maxratioDA)
    correctionratio=(zDsphere/zAsphere/maxratioDA)^0.5;
    zDsphere=zDsphere/correctionratio;
    zAsphere=zAsphere*correctionratio;
  end
  if (zAsphere/zDsphere>maxratioDA)
    correctionratio=(zAsphere/zDsphere/maxratioDA)^0.5;
    zDsphere=zDsphere*correctionratio;
    zAsphere=zAsphere/correctionratio;
  end
end
```

```
end
if (zsphere==0)
  maxshift=1;
  if (zDsphere<1E-5 && zAsphere<1E-5)
    if (improveDonor>improveAcceptor && improveDonor>0)
      shift=improveDonor*offtotal;
      if (shift>maxshift)
        shift=maxshift;
      end
      zDsphere=shift;
    end
    if (improveAcceptor>improveDonor && improveAcceptor>0)
      shift=improveAcceptor*offtotal;
      if (shift>maxshift)
        shift=maxshift;
      end
      zAsphere=shift;
    end
  else
    if (zDsphere<1E-5)
      shift=improveAcceptor*offtotal;
      if (shift>maxshift)
        shift=maxshift;
      end
      zAsphere=zAsphere+shift;
      if (zAsphere<0)
        zAsphere=0;
      end
    end
    if (zAsphere<1E-5)
      shift=improveDonor*offtotal;
```

```
        if (shift>maxshift)
          shift=maxshift;
        end
        zDsphere=zDsphere+shift;
        if (zDsphere<0)
          zDsphere=0;
        end
      end
    end
  end
end %end of function improve_Donor_Acceptor


function measure_error    %nested function within find_sphere
  closestminus=1000; furthestplus=0;
  nextminusin=0; nextminusout=1000;
  nextplusin=0; nextplusout=1000;
  offminus=0; offplus=0;
  improvex=0; improvey=0; improvez=0;
  nminus=0; nplus=0;
  fitquality=1; ndata=0;
  for i=1:20
    ioff(i)=0;
  end
  for i=1:nnegatives(solute)

[r,distx,disty,distz,realr]=parameter_distance(xsphere,ysphere,zsphere,zDsphere,zAspher
e,xnegative(solute,i),ynegative(solute,i),znegative(solute,i),zDnegative(solute,i),zAnegativ
e(solute,i),molvolume(negative(solute,i)),molvolume_solute(solute));
    if (r<closestminus)
      closestminus=r;
    end
    if (r<radius)
```

```
          nminus=nminus+1;
          if (r>nextminusin)
            nextminusin=r;
          end
          ioff(nminus)=negative(solute,i);
          off=radius-r;
          offminus=offminus+off;
          fitquality=fitquality*exp(-off);
          ndata=ndata+1;
          improvex=improvex+distx/realr/xfactor;
          improvey=improvey+disty/realr;
          improvez=improvez+distz/realr;
        else
          ndata=ndata+1;
        end
        if (r>radius && r<nextminusout)
          nextminusout=r;
        end
      end
      for i=1:npositives(solute)

[r,distx,disty,distz,realr]=parameter_distance(xsphere,ysphere,zsphere,zDsphere,zAspher
e,xpositive(solute,i),ypositive(solute,i),zpositive(solute,i),zDpositive(solute,i),zApositive(so
lute,i),molvolume(positive(solute,i)),molvolume_solute(solute));
        if (r>furthestplus)
          furthestplus=r;
        end
        if (r>radius)
          nplus=nplus+1;
          if (r<nextplusout)
            nextplusout=r;
          end
```

```
      ioff(nminus+nplus)=positive(solute,i);
      off=r-radius;
      offplus=offplus+off;
      fitquality=fitquality*exp(-off);
      ndata=ndata+1;
      improvex=improvex+-distx/realr/xfactor;
      improvey=improvey+-disty/realr;
      improvez=improvez+-distz/realr;
    else
      ndata=ndata+1;
    end
    if (r<radius && r>nextplusin)
      nextplusin=r;
    end
  end
  offtotal=offminus+offplus;
  fitquality=fitquality^(1/ndata);
end %end of function measure_error


function optimize_radius    %nested function within find_sphere
  % optimize radius at current position
  flagminus=0; flagplus=0;
  for loopradius=1:20
    measure_error;
    radiusold=radius;
    if (nnegatives(solute)==0)
      radius=furthestplus;
    elseif (nminus==0 || nplus==0)
      radius=(closestminus+furthestplus)/2;
    elseif (nplus>nminus)
      radius=nextplusout+0.0001;
```

```
  if (nplus-nminus==1)
    flagplus=1;
    if (flagminus==1)
      radius=nextplusout-0.0001;
    end
  end
elseif (nminus>nplus)
  radius=nextminusin-0.0001;
  if (nminus-nplus==1)
    flagminus=1;
    if (flagplus==1)
      radius=nextminusin+0.0001;
    end
  end
else
  if (nextplusout<nextminusout && nextplusin>nextminusin)
    radius=(nextplusout+nextplusin)/2;
  end
  if (nextplusout<nextminusout && nextplusin<nextminusin)
    radius=(nextplusout+nextminusin)/2;
  end
  if (nextplusout>nextminusout && nextplusin>nextminusin)
    radius=(nextminusout+nextplusin)/2;
  end
  if (nextplusout>nextminusout && nextplusin<nextminusin)
    radius=(nextminusout+nextminusin)/2;
  end
  break
end
if (radius-radiusold<0.00001 && radius-radiusold>-0.00001)
  break
```

```
      end
    end
  end %end of function optimize_radius

  function measure_error_symmetricradii    %nested function within find_sphere
    closestminus=1000; furthestplus=0;
    nextminusin=0; nextminusout=1000;
    nextplusin=0; nextplusout=1000;
    offminus=0; offplus=0;
    deltaoff=0;
    improvex=0; improvey=0; improvez=0;
    improveDonor=0; improveAcceptor=0;
    nminus=0; nplus=0;
    summinus_drdcsolid=0; sumplus_drdcsolid=0;
    fitquality=1; ndata=0;
    for i=1:20
      ioff(i)=0;loff(i)=0;
    end
    for i=1:nnegatives(solute)
[r,distx,disty,distz,realr]=parameter_distance(xsphere,ysphere,zsphere,zDsphere,zAspher
e,xnegative(solute,i),ynegative(solute,i),znegative(solute,i),zDnegative(solute,i),zAnegativ
e(solute,i),molvolume(negative(solute,i)),molvolume_solute(solute));
      cutoff=1/(1/radius+1/rnegative(solute,i));
[correction,volfraction2]=correction_for_concentration(concnegative(solute,i),volnegative(
solute,i));
      correction=correction*tempnegative(solute,i)/295;
      cutoff=cutoff*correction;
      if (r/correction>rnegative(solute,i))
        wishedr=1000;
      else
        wishedr=1/(correction/r-1/rnegative(solute,i));
```

```
        end
        if (correct4solid==1)
          if (correctc_solidfortemp==0)
            c_solid_local=c_solid;
          end
          if (correctc_solidfortemp==1)
            c_solid_local=c_solid*(Tm_solute(solute)-
tempnegative(solute,i))/(Tm_solute(solute)-295);
          end
          if (correctc_solidfortemp==2)
            c_solid_local=c_solid*(((Tm_solute(solute)-
tempnegative(solute,i))/(Tm_solute(solute)-295)+1)/2);
          end
          cutoff=cutoff-c_solid_local/volfraction2;
          if ((r+c_solid_local/volfraction2)/correction>rnegative(solute,i))
            wishedr=1000;
          else
            wishedr=1/(correction/(r+c_solid_local/volfraction2)-1/rnegative(solute,i));
          end
        end
        if (r<cutoff)
          nminus=nminus+1;
          if (wishedr>nextminusin)
            nextminusin=wishedr;
          end
          ioff(nminus)=negative(solute,i);
          % off is defined as the distance that the sphere would need to move keeping the
current radius. This is actually not correctly calculated (nor well-defined) in case of
usegeometricmean, but as off is only used to get an idea and is not used internally, this is
oke. (it is used to select the best solution though...)
          off=cutoff-r;
          loff(nminus)=off;
          offminus=offminus+off;
```

```
        fitquality=fitquality*exp(-off);

        ndata=ndata+1;

        deltacutoff=correction*rnegative(solute,i)^2/(radius+rnegative(solute,i))^2 ;
%=deltacutoff/deltaradius

        deltaoff=deltaoff+deltacutoff;

        improvex=improvex+distx/realr/xfactor;

        improvey=improvey+disty/realr;

        improvez=improvez+distz/realr;

        improveDonor=improveDonor-zAnegative(solute,i);

        improveAcceptor=improveAcceptor-zDnegative(solute,i);

        if (correct4solid==1)

dradiusdcsolid=correction/(volfraction2*(cutoff+c_solid_local/volfraction2)^2*(correction/
(cutoff+c_solid_local/volfraction2)-1/rnegative(solute,i))^2);

          summinus_drdcsolid=summinus_drdcsolid+dradiusdcsolid;

        end

      else

        ndata=ndata+1;

      end

      if (wishedr>radius && wishedr<nextminusout)

        nextminusout=wishedr;

      end

      if (wishedr<closestminus)

        closestminus=wishedr;

      end

    end

    for i=1:npositives(solute)

[r,distx,disty,distz,realr]=parameter_distance(xsphere,ysphere,zsphere,zDsphere,zAspher
e,xpositive(solute,i),ypositive(solute,i),zpositive(solute,i),zDpositive(solute,i),zApositive(so
lute,i),molvolume(positive(solute,i)),molvolume_solute(solute));

      cutoff=1/(1/radius+1/rpositive(solute,i));
```

```
[correction,volfraction2]=correction_for_concentration(concpositive(solute,i),volpositive(solute,i));

        correction=correction*temppositive(solute,i)/295;

        cutoff=cutoff*correction;

        if (r/correction>rpositive(solute,i))

          wishedr=1000;

        else

          wishedr=1/(correction/r-1/rpositive(solute,i));

        end

        if (correct4solid==1)

          if (correctc_solidfortemp==0)

            c_solid_local=c_solid;

          end

          if (correctc_solidfortemp==1)

            c_solid_local=c_solid*(Tm_solute(solute)-temppositive(solute,i))/(Tm_solute(solute)-295);

          end

          if (correctc_solidfortemp==2)

            c_solid_local=c_solid*(((Tm_solute(solute)-temppositive(solute,i))/(Tm_solute(solute)-295)+1)/2);

          end

          cutoff=cutoff-c_solid_local/volfraction2;

          if ((r+c_solid_local/volfraction2)/correction>rpositive(solute,i))

            wishedr=1000;

          else

            wishedr=1/(correction/(r+c_solid_local/volfraction2)-1/rpositive(solute,i));

          end

        end

        if (r>cutoff)

          nplus=nplus+1;

          if (wishedr<nextplusout)

            nextplusout=wishedr;
```

```
        end
        ioff(nminus+nplus)=positive(solute,i);
        off=r-cutoff;
        loff(nminus+nplus)=off;
        offplus=offplus+off;
        fitquality=fitquality*exp(-off);
        ndata=ndata+1;
        deltacutoff=correction*rpositive(solute,i)^2/(radius+rpositive(solute,i))^2;
        deltaoff=deltaoff+-deltacutoff;
        improvex=improvex+-distx/realr/xfactor;
        improvey=improvey+-disty/realr;
        improvez=improvez+-distz/realr;
        improveDonor=improveDonor+zApositive(solute,i);
        improveAcceptor=improveAcceptor+zDpositive(solute,i);
        if (correct4solid==1)

dradiusdcsolid=correction/(volfraction2*(cutoff+c_solid_local/volfraction2)^2*(correction/(cutoff+c_solid_local/volfraction2)-1/rpositive(solute,i))^2);
          sumplus_drdcsolid=sumplus_drdcsolid+dradiusdcsolid;
        end
      else
        ndata=ndata+1;
      end
      if (wishedr<radius && wishedr>nextplusin)
        nextplusin=wishedr;
      end
      if (wishedr>furthestplus)
        furthestplus=wishedr;
      end
    end
    offtotal=offminus+offplus;
    fitquality=fitquality^(1/ndata);
```

```
  if (furthestplus>200)
    furthestplus=200;
  end
  if (closestminus>200)
    closestminus=200;
  end
end %end of function measure_error_symmetricradii


function optimize_radius_symmetricradii    %nested function within find_sphere
  improve_Donor_Acceptor;
  % optimize radius at current position
  csolid_old=0;
  for loopsolid=1:100
    radiusold=0; offold=0;
    for loopradius=1:30
      measure_error_symmetricradii;
      radiusold2=radiusold;
      radiusold=radius;
      offold2=offold;
      offold=offtotal;
      if (nnegatives(solute)==0)
        radius=furthestplus;
      elseif (nminus==0 && nplus==0)
        radius=(closestminus+furthestplus)/2;
      elseif (deltaoff<0)
        if (nextplusout<nextminusout)
          radius=nextplusout+0.001;
        else
          radius=nextminusout+0.001;
        end
      elseif (deltaoff>0)
```

```
    if (nextplusin>nextminusin)
      radius=nextplusin-0.001;
    else
      radius=nextminusin-0.001;
    end
  end
  if (radius-radiusold2<0.00001 && radius-radiusold2>-0.00001)
    if (offold<offold2)
      radius=radiusold;
    end
    measure_error_symmetricradii;
    break
  end
end
if (correct4solid==1)
  csolid_old2=csolid_old;
  csolid_old=c_solid;
  if (nminus~=0 || nplus~=0)
    if (nminus==0)
      c_solid=c_solid-0.1;
    elseif (nplus==0)
      c_solid=c_solid+0.1;
    elseif (summinus_drdcsolid/nminus>sumplus_drdcsolid/nplus)
      c_solid=c_solid+0.1;
    else
      c_solid=c_solid-0.1;
    end
  end
  if (c_solid<0)
    c_solid=0;
  end
```

```
        if (c_solid==csolid_old2)
          c_solid=csolid_old;
          break
        end
      else
        break
      end
    end
  end %end of function optimize_radius_symmetricradii


  %actual start of function find_sphere:
  find_startsphere;
  xstart=xsphere; ystart=ysphere; zstart=zsphere; zDstart=zDsphere;
zAstart=zAsphere; rstart=radius;
  if (useorigHansen==1)
    optimize_radius;
  end
  if (useorigHansen==0)
    optimize_radius_symmetricradii;
  end
  %do some extra looping over a grid of starting positions to be able to select the best
solution...(grid is build from close to further from startsphere)
  nperfect=0;
  avgx=0; avgy=0; avgz=0; avgzD=0; avgzA=0; avgr=0; avgcsolid=0;
  minx=1000; miny=1000; minz=1000; minzD=1000; minzA=1000; minr=1000;
mincsolid=1000;
  maxx=-1000; maxy=-1000; maxz=-1000; maxzD=-1000; maxzA=-1000; maxr=-
1000; maxcsolid=-1000;
  best=1000;
  step=1;
  nsteps=4;
  for istep=0:nsteps
    for a=-istep*step:step:istep*step
```

```
for b=-istep*step+abs(a):step:istep*step-abs(a)
  stepc=2*(istep*step-abs(a)-abs(b));
  if (stepc==0)
    stepc=step;
  end
  for c=-istep*step+abs(a)+abs(b):stepc:istep*step-abs(a)-abs(b)
    xsphere=xstart+a;
    ysphere=ystart+b;
    zsphere=zstart+c;
    zDsphere=zDstart;
    zAsphere=zAstart;
    radius=rstart;
    if (useorigHansen==0)
      if (xsphere<0)
        xsphere=0;
      end
      if (ysphere<0)
        ysphere=0;
      end
      if (zsphere<0)
        zsphere=0;
      end
    end

    % Now find out how much these are off and minimize them.
    for loopposition=1:100
      if (useorigHansen==0)
        [zDsphere,zAsphere]=make_consistent_zDA(zsphere,zDsphere,zAsphere);
        improve_Donor_Acceptor;
      end
      if (useorigHansen==1)
```

```
  optimize_radius;
end    %measure_error is called inside these functions.
if (useorigHansen==0)
  optimize_radius_symmetricradii;
end


% optimize position (radius optimization is done after each positional step)
xsphereold=xsphere; ysphereold=ysphere; zsphereold=zsphere;
if (nplus+nminus>0)
  % step direction is (improvex,improvey,improvez); we now need to find out the step length by finding next plus or minus that will be wrongly included or excluded by moving the sphere in that direction
  stepsize=1000;
  for i=1:nnegatives(solute)
    [pardist,distx,disty,distz,realr]=parameter_distance(xsphere,ysphere,zsphere,zDsphere,zAsphere,xnegative(solute,i),ynegative(solute,i),znegative(solute,i),zDnegative(solute,i),zAnegative(solute,i),molvolume(negative(solute,i)),molvolume_solute(solute));
    distx=-distx; disty=-disty; distz=-distz;
    [correction,volfraction2]=correction_for_concentration(concnegative(solute,i),volnegative(solute,i));
    correction=correction*tempnegative(solute,i)/295;
    if (useorigHansen==0)
      cutoff=correction/(1/radius+1/rnegative(solute,i));
    else
      cutoff=radius;
    end
    if (correct4solid==1)
      if (correctc_solidfortemp==0)
        c_solid_local=c_solid;
      end
      if (correctc_solidfortemp==1)
```

```
          c_solid_local=c_solid*(Tm_solute(solute)-
tempnegative(solute,i))/(Tm_solute(solute)-295);

        end

        if (correctc_solidfortemp==2)

          c_solid_local=c_solid*(((Tm_solute(solute)-
tempnegative(solute,i))/(Tm_solute(solute)-295)+1)/2);

        end

        cutoff=cutoff-c_solid_local/volfraction2;

      end

      if (useorigHansen==0)

        % for some reason the nonspherical optimization does not work very well, so
we optimize as if it is still spherical

        vector2=xfactor*improvex^2+improvey^2+improvez^2;

        inproduct=xfactor*improvex*(xnegative(solute,i)-
xsphere)+improvey*(ynegative(solute,i)-ysphere)+improvez*(znegative(solute,i)-
zsphere);

        discriminant=inproduct^2-40*vector2*(pardist-cutoff);

        if (discriminant>=0)

          solution1=(inproduct-discriminant^0.5)/vector2;

          if (solution1>0 && solution1<stepsize)

            stepsize=solution1;

          end

        end

      end

      if (useorigHansen==1)

        vector2=xfactor^2*improvex^2+improvey^2+improvez^2;

        r2=xfactor^2*distx^2+disty^2+distz^2;

        inproduct=xfactor^2*distx*improvex+disty*improvey+distz*improvez;

        discriminant=4*inproduct^2-4*r2*vector2+4*vector2*cutoff^2;

        if (discriminant>=0)

          solution1=(2*inproduct-discriminant^0.5)/(2*vector2);

          solution2=(2*inproduct+discriminant^0.5)/(2*vector2);

          if (solution1>0 && solution1<stepsize)
```

```
          stepsize=solution1;
        end
      end
    end
  end
  for i=1:npositives(solute)

[pardist,distx,disty,distz,realr]=parameter_distance(xsphere,ysphere,zsphere,zDsphere,zA
sphere,xpositive(solute,i),ypositive(solute,i),zpositive(solute,i),zDpositive(solute,i),zAposit
ive(solute,i),molvolume(positive(solute,i)),molvolume_solute(solute));
    distx=-distx; disty=-disty; distz=-distz;

[correction,volfraction2]=correction_for_concentration(concpositive(solute,i),volpositive(so
lute,i));
    correction=correction*temppositive(solute,i)/295;
    if (useorigHansen==0)
      cutoff=correction/(1/radius+1/rpositive(solute,i));
    else
      cutoff=radius;
    end
    if (correct4solid==1)
      if (correctc_solidfortemp==0)
        c_solid_local=c_solid;
      end
      if (correctc_solidfortemp==1)
        c_solid_local=c_solid*(Tm_solute(solute)-
temppositive(solute,i))/(Tm_solute(solute)-295);
      end
      if (correctc_solidfortemp==2)
        c_solid_local=c_solid*(((Tm_solute(solute)-
temppositive(solute,i))/(Tm_solute(solute)-295)+1)/2);
      end
      cutoff=cutoff-c_solid_local/volfraction2;
    end
```

```
if (useorigHansen==0)

  % for some reason the nonspherical optimization does not work very well, so
we optimize as if it is still spherical;

  vector2=xfactor*improvex^2+improvey^2+improvez^2;

  inproduct=xfactor*improvex*(xpositive(solute,i)-
xsphere)+improvey*(ypositive(solute,i)-ysphere)+improvez*(zpositive(solute,i)-zsphere);

  discriminant=inproduct^2-40*vector2*(pardist-cutoff);

  if (discriminant>=0)

    solution2=(inproduct+discriminant^0.5)/vector2;

    if (solution2>0 && solution2<stepsize)

      stepsize=solution2;

    end

  end

end

if (useorigHansen==1)

  vector2=xfactor^2*improvex^2+improvey^2+improvez^2;

  r2=xfactor^2*distx^2+disty^2+distz^2;

  inproduct=xfactor^2*distx*improvex+disty*improvey+distz*improvez;

  discriminant=4*inproduct^2-4*r2*vector2+4*vector2*cutoff^2;

  if (discriminant>=0)

    solution1=(2*inproduct-discriminant^0.5)/(2*vector2);

    solution2=(2*inproduct+discriminant^0.5)/(2*vector2);

    if (solution2>0 && solution2<stepsize)

      stepsize=solution2;

    end

  end

end

end

vectorlength=(improvex^2+improvey^2+improvez^2)^0.5;

if (useorigHansen==0)

  stepsize=stepsize*2;

end
```

```
        if (vectorlength*stepsize>2)
          stepsize=2/vectorlength;
        end
        xsphere=xsphere+improvex*stepsize;
        ysphere=ysphere+improvey*stepsize;
        zsphere=zsphere+improvez*stepsize;
        if (useorigHansen==0)
          if (xsphere<0)
            xsphere=0;
          end
          if (ysphere<0)
            ysphere=0;
          end
          if (zsphere<0)
            zsphere=0;
          end
        end
      end
      if (xsphere-xsphereold<0.001 && xsphere-xsphereold>-0.001 && ysphere-
ysphereold<0.001 &&ysphere-ysphereold>-0.001 && zsphere-zsphereold<0.001 &&
zsphere-zsphereold>-0.001)
        break
      end
    end

    if (offtotal<best)
      best=offtotal;
      bestx=xsphere; besty=ysphere; bestz=zsphere; bestzD=zDsphere;
bestzA=zAsphere; bestr=radius; bestcsolid=c_solid;
      bestminus=nminus; bestplus=nplus;
      bestoff=offtotal; bestfit=fitquality;
      for i=1:20
```

```
        bestioff(i)=ioff(i);
      end
    end
    if (offtotal==0)
      nperfect=nperfect+1;
      avgx=avgcsolid+xsphere; avgy=avgcsolid+ysphere; avgz=avgcsolid+zsphere;
avgzD=avgcsolid+zDsphere; avgzA=avgcsolid+zAsphere; avgr=avgcsolid+radius;
avgcsolid=avgcsolid+c_solid;
      if (xsphere<minx)
        minx=xsphere;
      end
      if (xsphere>maxx)
        maxx=xsphere;
      end
      if (ysphere<miny)
        miny=ysphere;
      end
      if (ysphere>maxy)
        maxy=ysphere;
      end
      if (zsphere<minz)
        minz=zsphere;
      end
      if (zsphere>maxz)
        maxz=zsphere;
      end
      if (zDsphere<minzD)
        minzD=zDsphere;
      end
      if (zDsphere>maxzD)
        maxzD=zDsphere;
      end
```

```
          if (zAsphere<minzA)
            minzA=zAsphere;
          end
          if (zAsphere>maxzA)
            maxzA=zAsphere;
          end
          if (radius<minr)
            minr=radius;
          end
          if (radius>maxr)
            maxr=radius;
          end
          if (c_solid<mincsolid)
            mincsolid=c_solid;
          end
          if (c_solid>maxcsolid)
            maxcsolid=c_solid;
          end
        end
      end
    end
  end
end
xsphere=bestx; ysphere=besty; zsphere=bestz; zDsphere=bestzD; zAsphere=bestzA;
radius=bestr; c_solid=bestcsolid;
nminus=bestminus; nplus=bestplus;
offtotal=bestoff; fitquality=bestfit;
for i=1:20
  ioff(i)=bestioff(i);
end
if (offtotal==0)
```

```
      avgx=avgx/nperfect; avgy=avgy/nperfect; avgz=avgz/nperfect;

      avgzD=avgzD/nperfect; avgzA=avgzA/nperfect;

      avgr=avgr/nperfect; avgcsolid=avgcsolid/nperfect;

      xsphere=avgx; ysphere=avgy; zsphere=avgz; zDsphere=avgzD; zAsphere=avgzA;
radius=avgr; c_solid=avgcsolid;

      % make this average consistent again (note that only the radius is reoptimized, so the
resulting average strictly may actually not be a perfect solution):

      if (useorigHansen==0)

        [zDsphere,zAsphere]=make_consistent_zDA(zsphere,zDsphere,zAsphere);

      end

      if (useorigHansen==1)

        optimize_radius;

      end

      if (useorigHansen==0)

        optimize_radius_symmetricradii;

      end

    end

  end %end of function find_sphere


  function predict_solubility    %nested function within fit_sphere


[distance,distx,disty,distz,realr]=parameter_distance(fitresultx(solute),fitresulty(solute),fit
resultz(solute),fitresultzD(solute),fitresultzA(solute),solventx(solvent),solventy(solvent),sol
ventz(solvent),solventzD(solvent),solventzA(solvent),molvolume(solvent),molvolume_solu
te(solute));

    cutoff_local=1/(1/fitresultr(solute)+1/solventr(solvent))*temperature/295;

    if (c_solid_local==0 && distance<=cutoff_local)

      fprintf(outputfile,'Predicted partial solubility of %s in %s at %gK: soluble in all
concentrations\r\n',char(name_solute(solute)),char(name_solvent(solvent)),temperature);

    else

      factor=(distance+c_solid_local)/cutoff_local;

      SoverH5050=4*log(0.5);

      factor=factor*SoverH5050;

      molfraction=exp(factor);
```

```
      newvalue=factor;
      for ilocal=1:2000
        molfraction2=1-molfraction;
        factor=(distance+c_solid_local/molfraction2)/cutoff_local*SoverH5050;

factortest=(molfraction*log(molfraction)+molfraction2*log(molfraction2))/(molfraction*m
olfraction2);
        if (factortest-factor<0.001 && factor-factortest<0.001)
          concentration=1000*molfraction/(molfraction2*molvolume(solvent)) ; % in mol per
liter solvent!
          fprintf(outputfile,'Predicted partial solubility of %s in %s at %gK: %0.2g mol per
liter
solvent.\r\n',char(name_solute(solute)),char(name_solvent(solvent)),temperature,concent
ration);
          break
        end
        if (molfraction<=0.4)
          newvalue=newvalue+(factor-factortest)/2;
        end
        if (molfraction>0.4)
          newvalue=newvalue+(factor-factortest)/10*molfraction2;
        end
        if (newvalue>=0)
          newvalue=-0.1;
        end
        molfraction=exp(newvalue);
      end
    end
  end %end of function predict_solubility


  readinputs
% Do the actual fitting of the spheres for each solute in the testdata inputfiles:
  outputfile=fopen('output.txt','w');
  if (testdata_present==1)
```

```
  fprintf(outputfile,'\r\n');
  fprintf(outputfile,'\tfit results:\r\n');
  for solute=1:nsolutes
    fprintf(outputfile,'\r\n');
    fprintf(outputfile,'%s \r\n',char(name_solute(solute)));
    c_solid=0;
    fclose(outputfile);
    find_sphere;
    outputfile=fopen('output.txt','a');
    fitresultx(solute)=xsphere; fitresulty(solute)=ysphere; fitresultz(solute)=zsphere;
    fitresultzD(solute)=zDsphere; fitresultzA(solute)=zAsphere; fitresultr(solute)=radius;
    fitresultc_solid(solute)=c_solid;
    if (nperfect>0)
      fprintf(outputfile,'%i perfect fits have been found.\r\n',nperfect);
      fprintf(outputfile,'Range of the parameters within these perfect fits: %g-%g %g-%g
%g-%g %g-%g %g-%g %g-%g %g-
%g\r\n',minx,maxx,miny,maxy,minz,maxz,minzD,maxzD,minzA,maxzA,minr,maxr,mincsol
id,maxcsolid);
      fprintf(outputfile,'Averaged solution: %g %g %g %g %g %g
%g\r\n',avgx,avgy,avgz,avgzD,avgzA,avgr,avgcsolid);
      fprintf(outputfile,'Making this average consistent again gives the following
solution:\r\n');
    end
    fprintf(outputfile,'Hansen_parameters D, P, H, Hdonor, Hacceptor: %g %g %g %g
%g\r\n',xsphere,ysphere,zsphere,zDsphere,zAsphere);
    fprintf(outputfile,'Sphere radius: %g\r\n',radius);
    fprintf(outputfile,'Correction for melting solute: %g\r\n',c_solid);
    fprintf(outputfile,'Quality of the fit: nr of wrong_in, nr of wrong_out, fitquality: %i %i
%g\r\n',nminus,nplus,fitquality);
    if (fitquality<1)
      fprintf(outputfile,'Wrong_ins:');
      for i=1:nminus
        fprintf(outputfile,' %s',char(name_solvent(ioff(i))));
      end
```

```
        fprintf(outputfile,'\r\n');
        fprintf(outputfile,'Wrong_outs:');
        for i=nminus+1:nminus+nplus
          fprintf(outputfile,' %s',char(name_solvent(ioff(i))));
        end
        fprintf(outputfile,'\r\n');
      end
      fprintf(outputfile,'Used molar volume of solute and melting point: %g
%g\r\n',molvolume_solute(solute),Tm_solute(solute));
      fprintf(outputfile,'Technical output (formatted to be reused as input):\r\n');
      fprintf(outputfile,'%s %g %g %g %g %g %g %g %g
%g\r\n',char(name_solute(solute)),xsphere,ysphere,zsphere,zDsphere,zAsphere,radius,c_
solid,molvolume_solute(solute),Tm_solute(solute));
    end
  end


% Add the input from the fitresults files:
  for i=1:nprefits
    already=0;
    for j=1:nsolutes
      if (strcmp(prefitsolute(i),name_solute(j)))
        already=1;
      end
    end
    if (already==0)
      nsolutes=nsolutes+1;
      name_solute(nsolutes)=prefitsolute(i);
      solute=nsolutes;
      fitresultx(solute)=prefitx(i);
      fitresulty(solute)=prefity(i);
      fitresultz(solute)=prefitz(i);
      fitresultzA(solute)=prefitzA(i);
```

```
      fitresultzD(solute)=prefitzD(i);
      fitresultr(solute)=prefitr(i);
      fitresultc_solid(solute)=prefitc_solid(i);
      molvolume_solute(solute)=prefitvolume(i);
      Tm_solute(solute)=prefitTm(i);
    end
  end

% Make predictions using these fit results:
  if (predictions_present==1)
    correctin=0; correctout=0; wrongin=0; wrongout=0;
    fprintf(outputfile,'\r\n');
    fprintf(outputfile,'\tpredictions:\r\n');
    for iprediction=1:npredictions
      solutefound=0; solventfound=0;
      for k=1:nsolutes
        if (strcmp(predictsolute(iprediction),name_solute(k)))
          solute=k;
          solutefound=1;
        end
      end
      for k=1:nsolventstotal
        if (strcmp(predictsolvent(iprediction),name_solvent(k)))
          solvent=k;
          solventfound=1;
        end
      end
      if (solutefound==0)
        disp(['No data available for solute ',char(predictsolute(iprediction)),'; ignored.']);
      end
      if (solventfound==0)
```

```
disp(['No data available for solvent ',char(predictsolvent(iprediction)),'; ignored.']);

end

if (solutefound==1 && solventfound==1)

[distance,distx,disty,distz,realr]=parameter_distance(fitresultx(solute),fitresulty(solute),fitresultz(solute),fitresultzD(solute),fitresultzA(solute),solventx(solvent),solventy(solvent),solventz(solvent),solventzD(solvent),solventzA(solvent),molvolume(solvent),molvolume_solute(solute));

if (useorigHansen==1)

cutoff=fitresultr(solute);

end

if (useorigHansen==0)

cutoff=1/(1/fitresultr(solute)+1/solventr(solvent));

[correction,volfraction2]=correction_for_concentration(predictconc(iprediction),molvolume(solvent));

correction=correction*predicttemp(iprediction)/295;

if (correctc_solidfortemp==0)

c_solid_local=fitresultc_solid(solute);

end

if (correctc_solidfortemp==1)

c_solid_local=fitresultc_solid(solute)*(Tm_solute(solute)-predicttemp(iprediction))/(Tm_solute(solute)-295);

end

if (correctc_solidfortemp==2)

c_solid_local=fitresultc_solid(solute)*(((Tm_solute(solute)-predicttemp(iprediction))/(Tm_solute(solute)-295)+1)/2);

end

cutoff=cutoff*correction-c_solid_local/volfraction2;

end

if (distance<=cutoff)

fprintf(outputfile,'\r\n');

fprintf(outputfile,'Prediction: Solute %s dissolves in solvent %s at concentration %g mol/L and temperature
```

```
%gK.\r\n',char(name_solute(solute)),char(name_solvent(solvent)),predictconc(iprediction
),predicttemp(iprediction));

     if (strncmp(name_solvent(solvent),'mix_',4))

      splittedname=strsplit(char(name_solvent(solvent)),'_');

      imix=str2double(char(splittedname(2)));

      fprintf(outputfile,'(%s is ',char(name_solvent(solvent)));

      for i=1:npuresinmix(imix)

       fprintf(outputfile,'%g percent
%s',amountpureinmix(imix,i)*100,char(namepureinmix(imix,i)));

       if (i<npuresinmix(imix))

        fprintf(outputfile,' + ');

       end

      end

      fprintf(outputfile,')\r\n');

     end

     if (strcmp(predictresult(iprediction),'yes'))

      fprintf(outputfile,'The experimental result given in the input agrees with this
prediction: correctin.\r\n');

      correctin=correctin+1;

     end

     if (strcmp(predictresult(iprediction),'no'))

      fprintf(outputfile,'The experimental result given in the input does NOT agree with
this prediction: wrongin.\r\n');

      wrongin=wrongin+1;

     end

     temperature=predicttemp(iprediction);

     predict_solubility;

    end

    if (distance>cutoff)

     fprintf(outputfile,'\r\n');

     fprintf(outputfile,'Prediction: Solute %s does not dissolve in solvent %s at
concentration %g mol/L and temperature
%gK.\r\n',char(name_solute(solute)),char(name_solvent(solvent)),predictconc(iprediction
),predicttemp(iprediction));
```

```
        if (strncmp(name_solvent(solvent),'mix_',4))
          splittedname=strsplit(char(name_solvent(solvent)),'_');
          imix=str2double(char(splittedname(2)));
          fprintf(outputfile,'(%s is ',char(name_solvent(solvent)));
          for i=1:npuresinmix(imix)
            fprintf(outputfile,'%g percent
%s',amountpureinmix(imix,i)*100,char(namepureinmix(imix,i)));
            if (i<npuresinmix(imix))
              fprintf(outputfile,' + ');
            end
          end
          fprintf(outputfile,')\r\n');
        end
        if (strcmp(predictresult(iprediction),'no'))
          fprintf(outputfile,'The experimental result given in the input agrees with this
prediction: correctout.\r\n');
          correctout=correctout+1;
        end
        if (strcmp(predictresult(iprediction),'yes'))
          fprintf(outputfile,'The experimental result given in the input does NOT agree with
this prediction: wrongout.\r\n');
          wrongout=wrongout+1;
        end
        temperature=predicttemp(iprediction);
        predict_solubility;
      end
    end
  end
  fprintf(outputfile,'\r\n');
  fprintf(outputfile,'summary of predictions:\r\n');
  fprintf(outputfile,'# correct_in = %i\r\n',correctin);
  fprintf(outputfile,'# correct_out = %i\r\n',correctout);
```

```
    fprintf(outputfile,'# wrong_in = %i\r\n',wrongin);
    fprintf(outputfile,'# wrong_out = %i\r\n',wrongout);
  end
  fclose(outputfile);
end %end of function fit_sphere



function [correction,volfraction2] = correction_for_concentration(concentration,volume2)
  concentration2=1000/volume2;
  molfraction=concentration/(concentration+concentration2);
  molfraction2=1-molfraction;

SoverH=(molfraction*log(molfraction)+molfraction2*log(molfraction2))/(molfraction*molfr
action2);
  volfraction2=molfraction2;
  SoverH5050=4*log(0.5);
  correction=SoverH/SoverH5050;
end



function [zDout,zAout] = make_consistent_zDA(z,zD,zA)
  shift=(((zD-zA)^2+4*z^2)^0.5-zD-zA)/2;
  zDout=zD+shift;
  zAout=zA+shift;
end



function [distance,distx,disty,distz,realr] =
parameter_distance(xsphere,ysphere,zsphere,zDsphere,zAsphere,x,y,z,zD,zA,volume_solv
ent,volume_solute)
  global useorigHansen xfactor correct4solid correctc_solidfortemp
  if (useorigHansen==1)
    distx=xsphere-x;
```

```
    disty=ysphere-y;

    distz=zsphere-z;

    distance=(xfactor^2*distx^2+disty^2+distz^2)^0.5;

  end

  if (useorigHansen==0)

    distx=((xsphere^2+x^2)/2-xsphere*x)/20 ; % the division by 20 is to stay in the same
scale so that optimization magic numbers keep working.

    disty=((ysphere^2+y^2)/2-ysphere*y)/20;

    distz=((zDsphere*zAsphere+zD*zA-(zDsphere*zA+zAsphere*zD))/2)/20;

    signdistz=1;

    if (distz<0)

      signdistz=-1;

    end

% to keep directional information:

    if (x>xsphere)

      distx=-distx;

    end

    if (y>ysphere)

      disty=-disty;

    end

    distz=abs(distz);

    if (z>zsphere)

      distz=-distz;

    end

% distance is now a simple sum instead of spherical...

    distance=xfactor*abs(distx)+abs(disty)+signdistz*abs(distz);

  end

  realr=(distx^2+disty^2+distz^2)^0.5;

end


function terms = strsplit(s, delimiter)
```

```
%STRSPLIT Splits a string into multiple terms
%
%   terms = strsplit(s)
%       splits the string s into multiple terms that are separated by
%       white spaces (white spaces also include tab and newline).
%
%       The extracted terms are returned in form of a cell array of
%       strings.
%
%   terms = strsplit(s, delimiter)
%       splits the string s into multiple terms that are separated by
%       the specified delimiter.
%
%   Remarks
%   -------
%       - Note that the spaces surrounding the delimiter are considered
%         part of the delimiter, and thus removed from the extracted
%         terms.
%
%       - If there are two consecutive non-whitespace delimiters, it is
%         regarded that there is an empty-string term between them.
%
%   Examples
%   --------
%       % extract the words delimited by white spaces
%       ts = strsplit('I am using MATLAB');
%       ts <- {'I', 'am', 'using', 'MATLAB'}
%
%       % split operands delimited by '+'
%       ts = strsplit('1+2+3+4', '+');
%       ts <- {'1', '2', '3', '4'}
```

```
%
%       % It still works if there are spaces surrounding the delimiter
%       ts = strsplit('1 + 2 + 3 + 4', '+');
%       ts <- {'1', '2', '3', '4'}
%
%       % Consecutive delimiters results in empty terms
%       ts = strsplit('C,Java, C++ ,, Python, MATLAB', ',');
%       ts <- {'C', 'Java', 'C++', '', 'Python', 'MATLAB'}
%
%       % When no delimiter is presented, the entire string is considered
%       % as a single term
%       ts = strsplit('YouAndMe');
%       ts <- {'YouAndMe'}
%

%   History
%   -------
%       - Created by Dahua Lin, on Oct 9, 2008
%

%% parse and verify input arguments

assert(ischar(s) && ndims(s) == 2 && size(s,1) <= 1, ...
    'strsplit:invalidarg', ...
    'The first input argument should be a char string.');

if nargin < 2
    by_space = true;
else
    d = delimiter;
    assert(ischar(d) && ndims(d) == 2 && size(d,1) == 1 && ~isempty(d), ...
```

```
        'strsplit:invalidarg', ...
        'The delimiter should be a non-empty char string.');

    d = strtrim(d);
    by_space = isempty(d);
end

%% main

s = strtrim(s);

if by_space
    w = isspace(s);
    if any(w)
        % decide the positions of terms
        dw = diff(w);
        sp = [1, find(dw == -1) + 1];     % start positions of terms
        ep = [find(dw == 1), length(s)];  % end positions of terms

        % extract the terms
        nt = numel(sp);
        terms = cell(1, nt);
        for i = 1 : nt
            terms{i} = s(sp(i):ep(i));
        end
    else
        terms = {s};
    end

else
    p = strfind(s, d);
```

```
    if ~isempty(p)
        % extract the terms
        nt = numel(p) + 1;
        terms = cell(1, nt);
        sp = 1;
        dl = length(delimiter);
        for i = 1 : nt-1
            terms{i} = strtrim(s(sp:p(i)-1));
            sp = p(i) + dl;
        end
        terms{nt} = strtrim(s(sp:end));
    else
        terms = {s};
    end
end
end
```
